# Nucleophilic Substitution Reactions in the [B_3_H_8_]^−^ Anion in the Presence of Lewis Acids

**DOI:** 10.3390/molecules27030746

**Published:** 2022-01-24

**Authors:** Alexandra T. Shulyak, Evgeniy O. Bortnikov, Nikita A. Selivanov, Mikhail S. Grigoriev, Alexey S. Kubasov, Andrey P. Zhdanov, Alexander Y. Bykov, Konstantin Y. Zhizhin, Nikolai T. Kuznetsov

**Affiliations:** 1 Kurnakov Institute of General and Inorganic Chemistry, Russian Academy of Sciences, Leninskii Pr. 31, 119991 Moscow, Russia; GooVee@yandex.ru (N.A.S.); fobosax@mail.ru (A.S.K.); zhdanov@igic.ras.ru (A.P.Z.); bykov@igic.ras.ru (A.Y.B.); zhizhin@igic.ras.ru (K.Y.Z.); ntkuz@igic.ras.ru (N.T.K.); 2 Inorganic Chemistry Department, Lomonosov Institute of Fine Chemical Technologies, MIREA—Russian Technological University, Pr. Vernadskogo, 86, 119454 Moscow, Russia; 3 Weizmann Institute of Science, Organic Chemistry, 234 Herzl Street, Rehovot 7610001, Israel; bortnikovevol@gmail.com; 4 Frumkin Institute of Physical Chemistry and Electrochemistry, Russian Academy of Sciences, Leninskii Pr. 31-4, 119071 Moscow, Russia; grigoriev@ipc.rssi.ru

**Keywords:** boron, borohydrides, boranes, nucleophilic substitution, octahydrotriborate, cluster, Lewis acids

## Abstract

As a result of our study on the interaction between the octahydrotriborate anion with nucleophiles (Nu = THF, Ph_3_P, Ph_2_P-(CH_2_)_2_-PPh_2_ (dppe), Ph_3_As, Et_3_N, PhNH_2_, C_5_H_5_N, CH_3_CN, Ph_2_CHCN)) in the presence of a wide range of Lewis acids (Ti(IV), Hf(IV), Zr(IV), Al, Cu(I), Zn, Mn(II), Co(II) halides and iodine), a number of substituted derivatives of the octahydrotriborate anion [B_3_H_7_Nu] are obtained. It is found that the use of TiCl_4_, AlCl_3_, ZrCl_4_, HfCl_4_, CuCl and iodine leads to the highest product yields. In this case, it is most likely that the reaction proceeds through the formation of an intermediate [B_3_H_7_-HMXn_x_], which was detected by NMR spectroscopy. The structures of [Ph_3_P·B_3_H_7_] and [PhNH_2_·B_3_H_7_] were determined by X-ray diffraction.

## 1. Introduction

The interaction of the [B_3_H_8_]^−^ anion with nucleophiles in the presence of Lewis acids is a modern method for the preparation of its substituted derivatives [B_3_H_7_Nu] [1,2,3,4]. Special attention should be paid to the effect of Lewis acids on the reaction course. Halides that act as electrophilic initiators of the reaction have been reported: Hg(II) [5], Ti(IV), W(VI), Ge(IV) [6] and iodine [7]. Substituted derivatives of the octahydrotriborate anion [B_3_H_7_Nu] have long been of interest to researchers [8,9]. M[B_3_H_8_] compounds have been mentioned both for hydrogen storage and as ion conductors in future solid-state batteries [10]. The high content of hydrogen [11] in some adducts determines their use as chemical accumulators of hydrogen [7,12,13]. In addition, substituted derivatives of the octahydrotriborate anion can act as ligands in complexes of transition metals [14,15,16], which does not exclude their use in the preparation of boride coatings [17,18], as was previously shown for some complexes of the octahydrotriborate anion [19,20]. Substituted [B_3_H_8_]^−^ derivatives can also be considered as precursors to substituted higher boron clusters (e.g., B_5_H_9_, B_4_H_10_). The search for new ways of obtaining substituted derivatives of the [B_3_H_8_]^−^ anion and the improvement of existing methods of their synthesis would significantly expand the range of compounds suitable for solving relevant tasks [21].

**Scheme 1:**
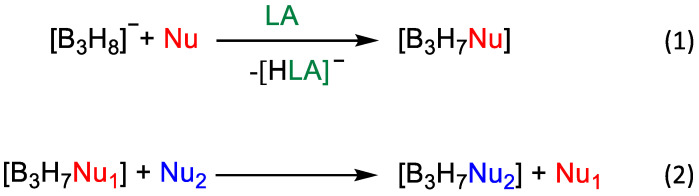
Reaction of nucleophilic substitution in the [B_3_H_8_]^−^ anion.

A convenient method for the synthesis of substituted derivatives of the octahydrotriborate anion is the direct interaction of the [B_3_H_8_]^−^ anion with nucleophiles in the presence of Lewis acids (LA), namely metal halides, iodine, boron fluoride and protic acids [6,7,22] (Scheme 1). The synthesis can be carried out both with an excess of nucleophile, and with its stoichiometric amount. Some compounds are obtained by the reaction of nucleophile metathesis: the weaker nucleophile (Nu1) (usually ethers) is displaced by the stronger one (Nu2) [6,7].

**Figure 1:**
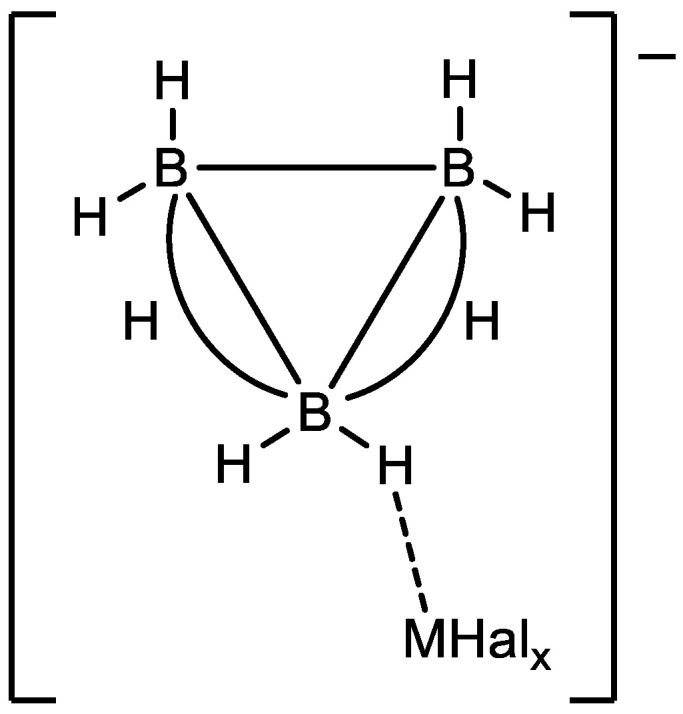
Possible structure of the transition complex [B_3_H_7_-HMHal_x_]^−^.

The study of the substitution reaction by NMR in the presence of various Lewis acids has made it possible, in the present work, to come closer to understanding the reaction processes. It is probable that the reaction is accompanied by the formation of the intermediate [B_3_H_7_-HMX_n_]^−^ (Figure 1). The stability of the complex is determined by the combination of [B_3_H_8_]^−^ nucleophilicity and the level of Lewis acidity.

## 2. Experimental Procedure

Synthetic experiments were carried out in an argon atmosphere in a sealed SPEX GB22M glove box with an automatic double H_2_ cleaning unit and two airlock chambers. The oxygen content did not exceed 20 ppm and the moisture content did not exceed 10 ppm. A propylene glycol bath with a cooled coil (using out-of-box cryostat) was used for cooling. Micro-volumes were measured using an electronic micropipette (Thermo Scientific Novus). 

### 2.1. Generation of [B_3_H_7_THF] 

Two equivalents of Lewis acid versus one equivalent of [Bu_4_N][B_3_H_8_] (50 mg, 0.177 mmol) were added to tetrahydrofuran (3 mL) while stirring. Immediate H_2_ release was observed. The reaction mixture was stirred for 2 h at room temperature, after which a sample was prepared for NMR spectroscopy studies. ^11^B NMR (THF, 298K, 96.32 MHz, ppm): −8.5 (m, J_BH_ = 38Hz, 2B), −13.4 (br, s, 1B) (THF·B_3_H_7_), −30.9 (m = 9, J_BH_ = 33Hz, [B_3_H_8_]^−^), −1.6 (q, J_BH_ = 33Hz, THF·BH_3_). Reactions with ZrCl_4_ and AlCl_3_ were also carried out under reflux for 2 h with the same weighed portions and observations.

### 2.2. Generation of [B_3_H_7_NCCH_3_]

(a) A solution of [Bu_4_N][B_3_H_8_] (30 mg, 0.106 mmol) in acetonitrile (2 mL) was cooled to −40 °C. Two equivalents of Lewis acid were added to the reaction solution. The mixture was cooled for 15 min and allowed to slowly warm to room temperature for 30 min; H_2_ gas release was observed. Reactions were also carried out at room temperature without cooling. NMR (CH_3_CN, 298 K, 96.32 MHz, ppm): ^11^B-{^1^H} δ: −7.6 (2B), −35.2 (1B), ^1^H (CD_2_Cl_2_, 298 K, 300 MHz, ppm): 1.97 (3H, CH_3_, s), 2.12–1.19 (7H, HB, br). IR (KBr), cm^−1^: ν(BH): 2516, 2446, 2372, 2340. Calcd./Found, %: C (29.83/29.87), H (12.51/12.56), N (17.31/17.29), B (40.27/40.21).

(b) A solution of [Bu_4_N][B_3_H_8_] (30 mg, 0.106 mmol) and CH_3_CN (55 μL, 0.106 mmol) in dichloromethane (2 mL) was cooled to −40 °C; AlCl_3_ (0.265 mmol) was added, while stirring. The mixture was cooled for 15 min and allowed to slowly warm to room temperature for 30 min; gas evolution was observed. NMR (CH_2_Cl_2_, 298 K, 100 MHz, ppm): ^11^B-{^1^H}, δ: −7.6 (2B), −35.2 (1B), ^1^H (CD_2_Cl_2_, 298 K, 300 MHz, ppm): 1.97 (3H, CH_3_, s), 2.12–1.19 (7H, HB, br).

### 2.3. Generation of [B_3_H_7_NCCHPh_2_]

A solution of [Bu_4_N][B_3_H_8_] (30 mg, 0.106 mmol) and Ph_2_CHCN (20 mg, 0.106 mmol) in dichloromethane (2 mL) was cooled to −40 °C and AlCl_3_ (0.265 mmol) was added, while stirring. The mixture was cooled for 15 min and allowed to slowly warm to room temperature for 30 min; H_2_ release was observed. ^11^B NMR (CH_2_Cl_2_, 298 K, 96.32 MHz, ppm): δ −8.0 (tm, 2B, J_BH_ = 130 Hz, B_4_H_10_), −7.65 (m, 2B, J_BH_ = 30 Hz Ph_2_CHCN·B_3_H_7_), −35.4 (s, 1B, Ph_2_CHCN·B_3_H_7_), −43.4 (dm, 2B, J_BH_ = 153Hz, B_4_H_10_).

### 2.4. Synthesis of [B_3_H_7_PPh_3_]

(a) Ph_3_P (46 mg, 0.177 mmol) was added, while stirring, to a solution of [Bu_4_N][B_3_H_8_] (50 mg, 0.177 mmol) in dichloromethane (3 mL). TiCl_4_ (38.7 μL, 0.352 mmol) was carefully added. Immediate H_2_ release was observed. The solution changed from being colourless to being brown, and a dark brown precipitate formed. The reaction mixture was stirred for 2 h at room temperature, after which a sample was prepared for NMR studies. When increasing the amount of nucleophile added to 0.354 mmol, the observations remained the same. 

(b) Ph_3_P (138 mg, 0.53 mmol) was added, while stirring, to a solution of [Bu_4_N][B_3_H_8_] (150 mg, 0.53 mmol) in dichloromethane (10 mL). A solution of I_2_ (134.5 mg) in the same solvent (10 mL) was added. Immediate H_2_ release was observed; the solution changed from being colourless to being brown, and a white precipitate formed. Subsequently, as the reaction proceeded, the discolouration of the reaction mixture was observed. The mixture was stirred for 2 h at room temperature, after which a sample was prepared for NMR analysis. 

To isolate the target adduct, the resulting reaction mixture was filtered, the filtrate was evaporated to a concentrated solution and chromatographic studies were performed (silica gel, dichloromethane as an eluent). Fractions collected with the target substance (Ph_3_P·BH_3_ and Ph_3_P·B_3_H_7_ mixtures) were poured into one flask, evaporated, dissolved in a mixture of hexane:dichloromethane with a ratio of 5:1 and chromatographic studies were performed again (silica gel, a mixture of hexane:dichloromethane = 5:1 as an eluent). The fractions with the target substance were collected in a flask and evaporated. As a result, 47 mg (0.156 mmol) of white powder of Ph_3_P·B_3_H_7_ was obtained. The yield was 29%. Crystals suitable for X-ray diffraction were obtained by the slow evaporation of a Ph_3_P·B_3_H_7_ solution in a hexane–dichloromethane mixture. ^11^B NMR (CD_2_Cl_2_, 298 K, 96.32 MHz, ppm): −17.4 (s, br, 2B, Ph_3_P·B_3_H_7_), −46.0 (d, br, 1B, J_BP_ = 94Hz, Ph_3_P·B_3_H_7_), ^1^H (CD_2_Cl_2_, 298 K, 300 MHz, ppm) δ: 7.68, 7.62, 7.54 (15H, Ph, m, J_HH_ = 36.2 Hz), 1.46–0.75 (7H, HB, br), ^31^P (CD_2_Cl_2_, 298 K, 121.49 MHz, ppm) δ: 22.12 (q, J_BP_ = 94Hz, [B_3_H_7_PPh_3_]). ^13^C-^1^H (CD_2_Cl_2_, 298 K, 75.49 MHz, ppm) δ: 132.9 ((3C, C(α)), 131.3 ((8C, C(β)), 128.3 ((3C, C(γ)), IR (cm^−1^): ν(BH): 2506, 2475, 2435, 2280. Calcd./Found, %: C, 71.64/71.67; H, 7.35/7.40; P, 10.26/10.20; B, 10.75/10.71.

### 2.5. Synthesis of [B_3_H_7_NEt_3_]

(a) [B_3_H_7_NEt_3_] was synthesised according to procedures a and b described for [B_3_H_7_Ph_3_P]. ^11^B NMR (CH_2_Cl_2_, 298 K, 96.32 MHz, ppm): −19.9 (tm, 2B, J_BH_ = 130Hz, B_4_H_10_), −22.4 (d, 4B, J_BH_ = 166 Hz, B_5_H_9_), −13.4 (q, J_BH_ = 99Hz, Et_3_N·BH_3_), −20.2 (s, br, 2B, Et_3_N·B_3_H_7_), −22.3 (s, br, 1B, Et_3_N·B_3_H_7_), −41.5 (dm, 2B, J_BH_ = 153Hz, B_4_H_10_), −53.2 (d, 1B, J_BH_ = 175 Hz, B_5_H_9_); −16.0 (s, br), −32.7 (t, J_BH_ = 149Hz), −65.3 (d, J_BH_ = 149Hz) unidentified substances.

(b) A better way for synthesis [B_3_H_7_NEt_3_]: A solution of 188.5 mg of AlCl_3_ (1.42 mmol) in tetrahydrofuran (15 mL) was added to a solution of [Bu_4_N] [B_3_H_8_] (200 mg, 0.71 mmol) in tetrahydrofuran (10 mL) placed in a 50 mL reaction vessel. The reaction mixture was stirred at reflux for 2 h, after which it was cooled to a temperature of −40 °C. After 5 min, 98.5 μL of Et_3_N (0.71 mmol) was added to the reaction mixture, the mixture was left at a constant temperature (−40 °C) for 30 min, while stirring, and slowly warmed to room temperature. The solution of the reaction mixture was colourless, and no precipitation was observed. Then, it was evaporated, and the precipitate was poured into dichloromethane (20 mL). The resulting solution was filtered, evaporated to 5–10 mL and chromatographic studies were performed (silica gel, dichloromethane as an eluent). Fractions with the target substance were poured into one flask and evaporated. As a result, 47 mg (0.334 mmol) of a colourless transparent liquid [B_3_H_7_Et_3_N] was produced. The yield was 47%. NMR: ^11^B-{^1^H} (CD_2_Cl_2_, 298 K, 96.32 MHz, ppm) δ: −19.9 (2B), −22.4 (1B); ^1^H (CD_2_Cl_2_, 298 K, 300 MHz, ppm) δ: 2.87 (6H, CH2, m = 4, J_HH_ = 7.2 Γц), 1.19 (9H, CH3, t, J_HH_ = 7.2 Hz), 1.64–0.48 (7H, HB, br), IR (thin layer), cm^−1^: ν(BH): 2499, 2452, 2425, 2310, 2254. Calcd./Found, %: C, 51.23/51.26; H, 15.76/15.79; N, 9.96/9.92; B, 23.05/23.01.

### 2.6. Generation of [B_3_H_7_ dppe] and [B_3_H_7_AsPh_3_]

The preparation of [B_3_H_7_dppe] and isolation of the target adduct were carried out. According to method b used for Ph_3_P, however, the ratio of the starting salt and nucleophile was 2:1. Finally, 39 mg (0.082 mmol) of a white powder of dppe·2B_3_H_7_ was obtained. The yield was 30%. NMR: ^11^B (CD_2_Cl_2_, 298 K, 96.32 MHz, ppm) δ: −15.6 (2B), −47.1 (1B, d, J_BP_ = 84 MHz); ^1^H (CD_2_Cl_2_, 298 K, 300 MHz, ppm) δ: 7.9, 7.71, 7.61, (20H, Ph, m), 2.4 (2H, HC, br d, J_PH_ = 2.8 Hz), 2.2 (2H, HC, br d, J_PH_ = 6.4 Hz), 1.41–0.72 (7H, HB, br). ^31^P (CD_2_Cl_2_, 298 K, 121.49 MHz, ppm) δ: 27.6 (q, J_BP_ = 84Hz, [B_3_H_7_dppe]). Calc./Found, %: C, 65.41/65.44; H, 8.02/8.08; B, 13.59/13.54.

The preparation of [B_3_H_7_AsPh_3_] and isolation of the target adduct were carried out according to method b used for Ph_3_P. The substance was not isolated: when attempting to perform chromatographic studies, the complete decomposition of the substance was observed on the column. NMR (CH_2_Cl_2_, 298 K, 100 MHz, ppm): ^11^B-{^1^H} (CD_2_Cl_2_, 298 K, 96.32 MHz, ppm) δ: −13.3 (2B), −42.2 (1B).

### 2.7. Synthesis of [B_3_H_7_Py] 

The preparation of [B_3_H_7_Py] was carried out according to method b described for Et_3_N·B_3_H_7_. As a result, 48.8 mg (0.412 mmol) of a colourless transparent liquid (Py·B_3_H_7_) was obtained. The yield was 58%. NMR: ^11^B-{^1^H} (CD_2_Cl_2_, 298 K, 96.32 MHz, ppm) δ: −9.6 (2B), −23.8 (1B); ^1^H (CD_2_Cl_2_, 298 K, 300 MHz, ppm) δ: 8.60 (2H, H(2,6), d, J_HH_ = 5.3 Hz), 8.02 (1H, H(4), t, J_HH_ = 7.6 Hz), 7.55 (2H, H(3,5), m), 2.47–1.32 (7H, HB, br); ^13^C-^1^H (CD_2_Cl_2_, 298 K, 75.49 MHz, ppm) δ: 147.8 (2C, C(2,6)), 141.3 (1C, C(4)), 125.9 (2C, C(3,5)). IR (thin layer), cm^−1^: ν(BH): 2499, 2437, 2322. Calcd./Found, %: C, 50.64/50.62; H, 10.20/10.23; N, 11.81/11.75; B 27.35/27.31.

### 2.8. Synthesis of [B_3_H_7_NH_2_Ph] 

[B_3_H_7_ NH_2_Ph] was prepared according to method b described for Et_3_N·B_3_H_7_. As a result, 45.6 mg (0.344 mmol) of white powder (Py·B_3_H_7_) was obtained. The yield was 48%. NMR: ^11^B (CD_2_Cl_2_, 298 K, 96.32 MHz, ppm) δ: −8.9 (m, 2B, J_BH_ = 130Hz), −26.7 (s, 1B); Response to Reviewer 1 Comments; ^13^C-^1^H (CD_2_Cl_2_, 298 K, 75.49 MHz, ppm) δ: 139.3 (C_quat_, C(1)), 130.3 (2C, C(3,5)), 128.5 (1C, C(4)), 121.9 (2C, C(2,6)). IR (KBr, cm^−1^): ν(BH): 2505, 2431, 2322. Calcd./Found, %: C, 54.34/54.36; H, 10.64/10.68; N, 10.56/10.51; B, 24.46/24.42. Crystals suitable for X-ray diffraction studies were obtained by the slow evaporation of a PhNH_2_·B_3_H_7_ solution in a hexane–dichloromethane mixture.

## 3. Results and Discussion

### 3.1. Interaction with Lewis Acids

The molar ratio of the initial amounts of tetrabutylammonium octahydrotriborate and electrophile in this work was 1:2 (1:1 in the case of diiodine). This ratio was established experimentally. If the lesser ratio is used, the reaction does not proceed completely. It should be noted that [7] describes the synthesis of [B_3_H_7_DME] in dimethoxyethane in the presence of iodine in a 2:1 ratio with the initial salt, but, in the case of the present study, the highest yield was observed at the 1:1 ratio.

(1) $$2{\left[B_{3}H_{8}\right]}^{-}+I_{2}+\mathrm{DME}\underset{\mathrm{DME}}{\to }2\left[B_{3}H_{7}\mathrm{DME}\right]+H_{2}+2I^{-}$$

Dimethoxyethane and nucleophiles, such as tetrahydrofuran and acetonitrile, are liquids that allow one to perform the synthesis of substituted derivatives directly in the nucleophiles. A significant number of nucleophiles, however, are solids and, in this case, weaks nucleophilic haloalkanes (dichloromethane, dichloroethane) are usually used as a solvent. In the present work, substituted products were synthesised in dichloromethane at a 1:1 ratio of the molar amounts of nucleophile to the initial salt.

**Scheme 2:**
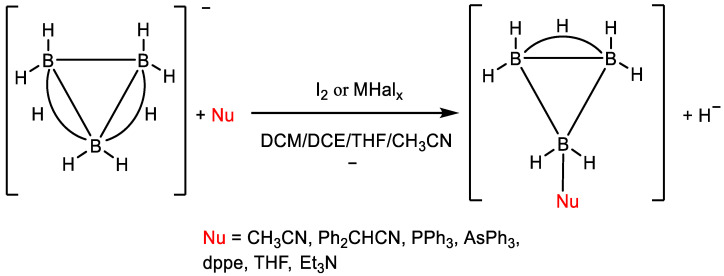
Substitution reaction in the octahydrotriborate anion.

The reaction (Scheme 2) gives a substituted product [B_3_H_7_Nu], where Nu = Ph_3_P, dppe, Ph_3_As, Et_3_N, CH_3_CN, Ph_2_CHCN. 

When the reaction was carried out with a stoichiometric amount of nucleophiles, the ^11^B NMR spectrum in dichloromethane showed signals from [B_4_H_10_] [23] and substituted products of monoborane [BH_3_Nu] (Figure S1). Since the reactions of the formation of the substituted product and the cleavage of the boron cage of the unstable [B_3_H_7_], in this case, can proceed in parallel [23,24], a part of the nucleophile is spent on stabilising the [BH_3_Nu] molecules formed because of the cleavage. As a result, in the absence of a nucleophile, the octahydrotriborate residue reacts with a Lewis acid to form tetraborane-10. It seems that the kinetics of the reaction are also affected by the nature of the nucleophile and the steric factor. Thus, in the reaction between [B_3_H_8_]^−^ and acetonitrile in dichloromethane in the presence of AlCl_3_, 2 h after the beginning of the reaction, the formation of a substituted product was observed and signals from tetraborane were absent; under the same conditions in the reaction with diphenylacetonitrile, signals of tetraborane, other condensation products and a small amount of the substituted derivative were observed after 2 h (Figures S2 and S3).

The synthesis of substituted derivatives in organic solvents with nucleophilic properties (in acetonitrile and tetrahydrofuran, respectively) leads to the complete absence of [B_4_H_10_] signals in the spectra of reaction mixtures. The highest yield of the product [B_3_H_7_THF] was achieved at 66 °C. On heating, there is an increase in the number of by-products, namely THF BH_3_, HB(OR)_2_, B(OR)_3_ [22]; however, their amount in the mixture remains rather low. It is known that, in the presence of LiAlH_4_, AlCl_3_ cleaves tetrahydrofuran to n-butyl alcohol, which reacts with octahydrotriborate to give HB(OR)_2_, B(OR)_3_ adducts [24].

In the case of acetonitrile, heating not only has no effect on the yield of the substituted product [B_3_H_7_NCCH_3_], but also promotes the formation of a significant number of by-products [25,26]. Therefore, a high yield can be achieved by cooling the reaction mixture to −40 °C and slowly heating to room temperature. It should be noted that, in conditions of an excess of nucleophile, the formation of products of cleavage and condensation of [B_3_H_7_] fragments were not observed either at room temperature or at an increased temperature; these products, in some cases, can occur during the interaction of a substituted product with an excess of nucleophiles [27,28,29].

Thus, the choice of a solvent is usually determined by the state of aggregation of the nucleophile and its nature. In the case of the present work, carrying out the reaction in weak nucleophilic and non-nucleophilic solvents made it possible to set the desired ratio of the nucleophile and the octahydrotriborate anion.

### 3.2. Metathesis of Nucleophiles

Some substituted products are more convenient to obtain by nucleophile metathesis [27,30,31]. It has been previously shown that [B_3_H_7_DME] obtained by the interaction between tetrabutylammonium octahydrotriborate and iodine in monoglyme subsequently reacts with ammonia to form the NH_3_·B_3_H_7_ adduct [7]. Similar results have been obtained for a number of phosphine-substituted adducts in tetrahydrofuran [6]. In the present work, most attention was paid to the further development of this technique by the synthesis of some N-substituted derivatives of the octahydrotriborate anion with the participation of metal halides.

Guided by the literature data [32], the synthesis was carried out in tetrahydrofuran through the formation of [B_3_H_7_THF]. Alkylamines, nitrogen-containing heterocycles, aniline and substituted phosphines are much stronger nucleophiles than tetrahydrofuran; therefore, they easily displace it. In this case, no further reaction of the substituted product with the nucleophile and the formation of condensation products was observed. The phosphine adducts were obtained in the same way, but a significant decrease in the product yield was observed.

(2) $$\begin{array}{ccc} \left[{\mathrm{Bu}}_{4}N\right]\left[B_{3}H_{8}\right]+{\mathrm{AlCl}}_{3}+\mathrm{THF} & \overset{\mathrm{THF}}{\underset{66^{\circ }C,2h}{\to }} & \mathrm{THF}\cdot B_{3}H_{7}\overset{\mathrm{Nu}}{\underset{-40^{\circ }C}{\to }}\mathrm{Nu}\cdot B_{3}H_{7}\\ \mathrm{Nu}={\mathrm{Et}}_{3}N,\mathrm{Py},{\mathrm{PhNH}}_{2} & -{\left[{\mathrm{HAlCl}}_{3}\right]}^{-} & \\ & -{\left[{\mathrm{Bu}}_{4}N\right]}^{+} & \end{array}$$

To increase the yield of the required substituted product, the reaction was carried out at low temperatures (from −40 °C to −30 °C), since, with an increase in the reaction temperature, for example, up to 25 °C, the processes of cleavage of the boron cluster become more pronounced [31]. In the present case, a significant amount of monoborane adduct was observed among the products and the initial tetrahydrofuran adduct underwent incomplete conversion. The rate of the metathesis reaction at −40 °C remains high, and upon further heating of the reaction mixture to room temperature, the composition of the mixture was unchanged, due to the complete binding of the added nucleophile to the borane fragments (at equimolar ratios).

Thus, it was shown that the conversion of the starting materials in the reaction of salts of the octahydrotriborate anion with metal halides in tetrahydrofuran could be significantly increased by heating; this interaction can be a convenient first step in the “one-pot” synthesis of a number of substituted derivatives of the octahydrotriborate anion. The addition of a nucleophile to the resulting reaction mixture at low temperatures made it possible to obtain the desired product, which was demonstrated for some N-nucleophiles and their corresponding derivatives.

### 3.3. Influence of Lewis Acids on the Evolution of the Reaction

**Scheme 3:**
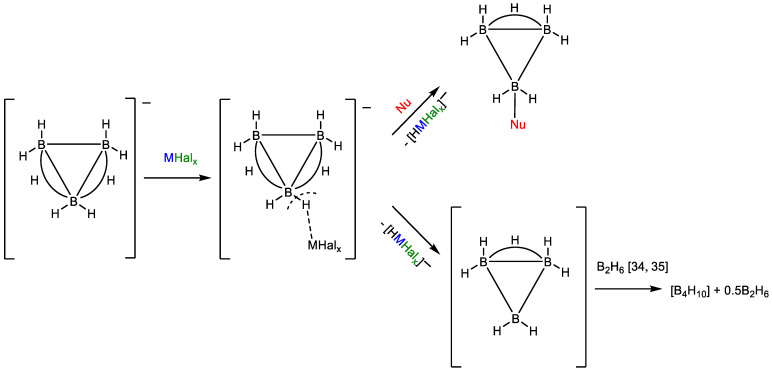
Possible scheme of the substitution reaction in the octahydrotriborate anion.

The interaction of the [B_3_H_8_]^−^ anion with nucleophiles occurs by the mechanism of electrophilic-induced nucleophilic substitution (EINS), similar to [B_10_H_10_]^2−^ and [B_12_H_12_]^2−^ [2] (Scheme 3). In the present study, it was found that the addition of a Lewis acid to a hydride ion at the first stage of the reaction resulted in the formation of an intermediate [B_3_H_7_-HX_n_]^−^, similar to the complexes reported in [33]. This complex was manifested in the ^11^B spectra as a weakly resolved signal, instead of a multiplex from the octahydrotriborate ion shifted towards a low field, the intensity of which decreased during the reaction. At the same time, the intensity of signals from the monosubstituted product increased. The HMX_n_^−^ molecule possesses nucleophilic properties with respect to [B_3_H_7_] and, in the presence of a stronger nucleophile, it is replaced, with the formation of [B_3_H_7_Nu]. When the nucleophile is absent, the HMX_n_^−^ molecule is split off; however, it is unable to stabilize [B_3_H_7_] because of its almost negligible nucleophilicity. The remaining [B_3_H_7_] undergoes cleavage and condensation, with the formation of tetraborane-10, according to the mechanism proposed by R. Schaeffer [34,35] using an isotopic label method. The authors obtained [B_4_H_10_] through the reaction of [B_3_H_8_]^−^ with hydrogen chloride as an electrophile. According to this mechanism during the reaction, the [B_2_H_6_] forms and reacts with the [B_3_H_7_] molecule to produce [B_4_H_10_] and 0.5B_2_H_6_.

(3) $${}^{10}B_{3}H_{7}{+}^{11}B_{2}H_{6}{\overset{}{\to }}^{10}B_{3}{}^{11}{\mathrm{BH}}_{10}+0.5^{11}B_{2}H_{6}$$

The path of the substitution reaction is most clearly seen when using copper(I) chloride as an electrophile. In the ^11^B and ^11^B {^1^H} spectra (Figures S4 and S5), the signal of the [B_3_H_7_-H-CuCl]^−^ complex can be clearly seen at −36.2 ppm, while [B_3_H_8_] corresponds to the signal at −30.9 ppm [36]. These data agree with the literature sources in which the structure of such a complex calculated by the DFT method [35] has been reported. This complex was quite stable; however, in an excess of acetonitrile, 30 min after the beginning of the reaction, a low-intensity signal began to appear at −7.5 ppm, corresponding to unsubstituted boron atoms of the resulting [B_3_H_7_NCCH_3_]. A few hours after the beginning of the reaction, the intensity of the signal from the complex decreased significantly until it disappeared, while the intensity of signals from the substituted product increased considerably. In the present case, in dichloromethane in the presence of a stoichiometric amount of acetonitrile, the complex did not decompose, and no formation of substituted products was observed.

Transition complexes have also been detected in reactions with zinc(II), cobalt(II) and manganese(II) chlorides [37,38,39]; however, for them, the metathesis of nucleophiles and the formation of a monosubstituted product practically did not occur, even in a solution of acetonitrile or tetrahydrofuran.

Metal halides’ hydride–ion affinity plays a significant role in the EINS reaction. At the same time, molecules of the nucleophilic solvents and metal halides can form complexes, which are also Lewis acids. Unfortunately, there are no data to be observed from the present study relating to Lewis acids’ hydride–ion affinity. Thus, in order to establish a complete and accurate pattern of behaviour for metal halides, as well as their complexes with solvent molecules as electrophiles in the substitution reaction in dichloromethane, acetonitrile, tetrahydrofuran, etc., it is necessary to carry out significant detailed studies using quantum chemical calculations, which is a separate, and major, task.

It is, however, now possible to qualitatively assess some regularity in terms of the behaviour of Lewis acids in the substitution reaction in various solvents. There is a time of the substituted products formation in the reaction of [B_3_H_8_]^−^ with CH_3_CN in the presence of various Lewis acids in dichloromethane and acetonitrile and with THF in THF is provided in Table S1.

The nature of the Lewis acid determines the electrophilicity of the boron atom. The stronger the Lewis acid, the more it increases the leaving group abilities of the [HLA]^−^ complex and electrophilicity of the boron atom that the nucleophile attacks. It is also necessary to take into account the effect of the amount of nucleophile used.

Thus, Ti(IV), Zr(IV), Hf(IV) and Al(III) chlorides are very strong electrophiles, either in dichloromethane or in acetonitrile and tetrahydrofuran, so the octahydrotriborate anion interaction with them in the presence of a nucleophile always leads to rapid substitution (Table S1). In the present case, the complexes formed in a nucleophilic solvent with solvent molecules did not have less electrophilicity than the metal halides.

Zn(II), Co(II) and Mn(II) chlorides are weak electrophiles, regardless of the solvent used and the amount of nucleophile. In these cases, the reaction stops at the formation of a complex, which can subsequently be destroyed due to an intramolecular redox reaction.

Copper(I) chloride in dichloromethane is a weak electrophile, such as Zn(II), Co(II) and Mn(II) chlorides. At the same time, in acetonitrile and tetrahydrofuran, the transition complex formed after 30 min generated a substituted product and after 12 h only the substituted product was observed.

### 3.4. NMR Data

The ^11^B NMR spectra of the Nu·B_3_H_7_ adducts (Nu = THF, Ph_3_P, Ph_2_P-(CH_2_)_2_-PPh_2_ (dppe), Ph_3_As, Et_3_N, PhNH_2_, C_5_H_5_N, CH_3_CN and Ph_2_CN) show two signals with an integrated intensity ratio of 2:1 (Figures S1–S6, S10, S12 and S14) [40]. The chemical shifts of the signals of boron atoms in adducts Py·B_3_H_7_ (−9.6 ppm; −23.8 ppm) (Figure S12) and PhNH_2_·B_3_H_7_ (−8.9 ppm; −26.7 ppm) (Figure S8) are quite similar; a significant shift of the signal of boron atoms of unsubstituted positions to the weak field relative to the signal of the boron atom of the substituted position can be observed. At the same time, for Et_3_N·B_3_H_7_ (−19.9 ppm; −22.4 ppm) (Figure S14), the situation is slightly different: the positions of the signals correlate with the data for Me_3_N·B_3_H_7_ [41], but the separation of signals from the boron atoms of different types can be clearly observed.

In addition, one broadened signal of hydrogen atoms in the boron fragment can be seen in the ^1^H NMR spectra for adducts, which indicates the occurrence of intramolecular hydrogen exchange processes (Figures S8, S11, S13 and S15). Chemical shifts of H_C_ signals in the Et_3_N (Figure S12) molecule differ from the chemical shifts of protons in the composition of non-bonded nucleophile molecules; in most cases, a shift of signals to the weak field is observed upon binding.

The ^31^P NMR spectra of the Nu·B_3_H_7_ adducts (Nu = Ph_3_P, Ph_2_P-(CH_2_)_2_-PPh_2_ (dppe)) show one signal at δ = 22.12 (27.6 for [B_3_H_7_dppe]) ppm (Figure S9). At the same time, the ^31^P{^1^H} NMR spectra of the pure PPh_3_, for example, show one signal at δ = −5.59 ppm.

### 3.5. X-ray Diffraction Data

**Figure 2:**
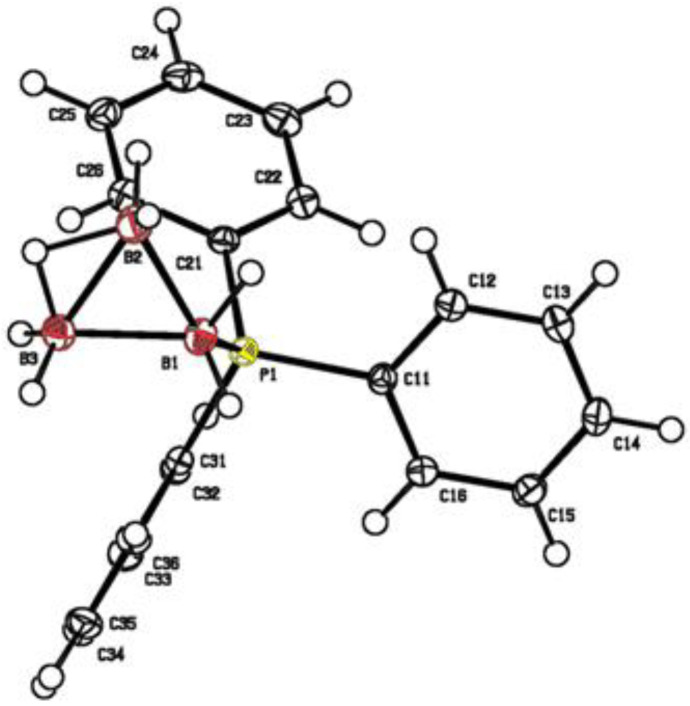
Structure of Ph_3_P·B_3_H_7_ determined by X-ray diffraction (temperature displacement ellipsoids are shown with a 50% probability level).

**Figure 3:**
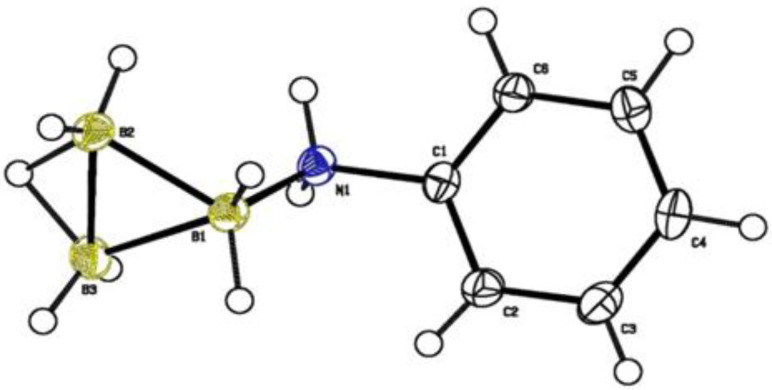
Structure of PhNH_2_·B_3_H_7_ determined by X-ray diffraction (temperature displacement ellipsoids are shown with a 50% probability level).

Figure 2 and Figure 3 show a general view of the structures Ph_3_P·B_3_H_7_ and PhNH_2_·B_3_H_7_. The main crystallographic data and values of selected bond lengths and bond angles are shown in Tables S2–S5. A noticeable similarity of the structure of the triphenylphosphine adduct of triborane with the structures given, for example, for H_3_N·B_3_H_7_ [7,42], Ph_3_PCH_2_·B_3_H_7_ [43] and [B_3_H_7_NCS]^−^ [44], can be observed: a three-centred two-electron B–H–B bond, as well as a half-bridge bond on one of the adjacent sides of the boron triangle, can be seen.

The structural data obtained for Ph_3_P·B_3_H_7_ and PhNH_2_·B_3_H_7_ (Figure 2 and Figure 3) largely correlate with the data presented for other triborane adducts. The B–B bond lengths tend to decrease with an increase in the contribution of the bridging of the BHB fragment. For example, in structure Ph_3_P·B_3_H_7_ d(B2–B3) (bridging bond B2–H23–B3) < d (B1–B2) (half-bridging bond B1–H1A–B2) < d(B1–B3) (no BHB interactions) (Table 1).

In the structure of Ph_3_P·B_3_H_7_, the hydrogen atom H23 was slightly more displaced towards the boron atom B3, which did not participate in the bridge interactions. The B1–H1A bond length of the bridged fragment correlated with similar values of the terminal B–H bonds in this structure. The B2–H1A distance was 1.65(2) Å, and this value was found to be much larger than the BH bond lengths of the bridging fragments; at the same time, it was noticeably shorter than the identical bond lengths, for example, in the structure of [B_3_H_7_NCBH_3_]^−^ (2.08(4) Å and 1.95(4) Å). The position of the H1A and H1B hydrogen atoms was distorted relative to the ideal symmetric position because of the presence of the B2–H1A interaction. It should also be noted that the hydrogen atom H1A lay in the plane of the boron triangle, as the bridging atom H23, (the torsion angles H23–B3–B1–B2 and H1A–B1–B2–B3 were 0.9(9)° and −179(1)°, respectively). The P1–B1 bond lengths, C–P bond lengths and C–P–B bond angles were in significant agreement with the data given for the monoborane Ph_3_P·BH_3_ adduct [45].

In the structure of PhNH_2_·B_3_H_7_, the B3–H1A and B2–H1B distances were 1.97(3) and 2.12 (3) Å, respectively. It can be argued that the bridging nature of the bond, which can be observed, for example, for H_3_N·B_3_H_7_ [7,32], Ph_3_PCH_2_·B_3_H_7_ [44], [B_3_H_7_NCS]^−^ and [B_3_H_7_NCSe]^−^ [44], was rather absent in the case of PhNH_2_·B_3_H_7_. The B1–N1 distance in the structure of PhNH_2_·B_3_H_7_ is in agreement with the data given for NH_3_·B_3_H_7_ (1.585(2) Å) [7]. The phenyl group and the borohydrogen fragment at the ends of the B1–N1 bond were faced in opposite directions, which corresponds to the least sterically hindered conformation. The N1–C1 distance was significantly increased compared to its value in the structure of free aniline, which was 1.478(2) Å for PhNH_2_·B_3_H_7_ versus 1.388(2) Å for the free ligand [46], due to the participation of the free electron pair of nitrogen in the binding with the borohydride fragment. It is also worth noting that the angle between the planes of the boron triangle and the phenyl fragment was 28.3°.

## 4. Conclusions

The interaction of the octahydrotriborate anion with nucleophiles in the presence of Lewis acids is a good way to obtain its monosubstituted derivatives. In the present case, the choice of suitable conditions was determined by the stability of the transition complex [B_3_H_7_-HMX_n_]^−^ formed during the reaction and the difference between the donor strength of the HMHalx^−^ molecule and the nucleophile, which is influenced by both the nature of the Lewis acid and the nature and amount of the nucleophile itself.

## Figures and Tables

**Table 1:** The structural data obtained for Ph_3_P·B_3_H_7_ and PhNH_2_·B_3_H_7__._

Bond	Ph_3_P·B_3_H_7_	PhNH_2_·B_3_H_7_
B–P(N)	1.926(1)	1.608(3)
B1–B2(B3)	1.804(2) 1.821(2)	1.847(3) 1.830(3)
B2–B3	1.779(2)	1.719(4)
B2–H_br_	1.33(2)	1.32(4)
B3–H_br_	1.17(2)	1.27(4)
av. B1–H	1.10	1.12
av. B2(3)–H_term_	1.11	1.12

## Data Availability

The data (spectroscopy data, X-ray data, and ESI data) used to support the findings of this study are included within the article.

## Supplementary Materials

The following supporting information can be downloaded. The supporting information file includes the figures of NMR spectra and tables of X-ray diffraction that were not provided in manuscript. Figure S1: ^11^B NMR spectrum of a mixture of TBA[B_3_H_8_], PPh_3_ and TiCl_4_ in CH_2_Cl_2_ 30 min after the beginning of the reaction. [B_3_H_7_PPh_3_] (δ −17.61 ppm, −45.76 ppm) at 298 K; Figure S2: ^11^B NMR spectrum of a mixture of TBA[B_3_H_8_], CH_3_CN and AlCl_3_ in CH_2_Cl_2_ 2h after the beginning of the reaction. [B_3_H_7_NCCH_3_] (δ −7.45 ppm, −35.25 ppm) at 298 K; Figure S3: ^11^B NMR spectrum of a mixture of TBA[B_3_H_8_], Ph_2_CHCN and AlCl_3_ in CH_2_Cl_2_ 2h after the beginning of the reaction. [B_3_H_7_NCCH_2_Ph] (δ −7.65 ppm, −35.44 ppm), [B_4_H_10_] (δ −8.01 ppm, −43.46 ppm) at 298 K; Figure S4: ^11^B-^1^H NMR spectrum of a mixture of TBA[B_3_H_8_] and CuCl in CD_3_CN 30 min after the beginning of the reaction. [B_3_H_7_NCCH_3_] (δ −7.58 ppm, −35.20 ppm), [BH_3_NCCH_3_] (δ −25.8 ppm), [B_3_H_7_HCuCl]^−^ (δ −36.20 ppm) at 298 K; Figure S5: ^11^B NMR spectrum of a mixture of TBA[B_3_H_8_] and CuCl in CD_3_CN 30 min after the beginning of the reaction. [B_3_H_7_NCCH_3_] (δ −7.58 ppm, −35.20 ppm), [BH_3_NCCH_3_] (δ −25.8 ppm), [B_3_H_7_HCuCl]^−^ (δ −36.20 ppm) at 298 K; Figure S6: ^11^B-^1^H NMR spectrum of crystal solution [B_3_H_7_PPh_3_] in CD_2_Cl_2_ at 298 K; Figure S7: ^1^H NMR spectrum of crystal solution [B_3_H_7_PPh_3_] in CD_2_Cl_2_ at 298 K; Figure S8: ^11^B-^1^H NMR spectrum of crystal solution [B_3_H_7_NH_2_Ph] in CD_2_Cl_2_ at 298 K; Figure S9: ^31^P NMR spectrum of crystal solution [B_3_H_7_PPh_3_] in CD_2_Cl_2_ at 298 K; Figure S10: ^11^B-^1^H NMR spectrum of crystal solution [B_3_H_7_NH_2_Ph] in CD_2_Cl_2_ at 298 K; Figure S11: ^1^H NMR spectrum of crystal solution [B_3_H_7_NH_2_Ph] in CD_2_Cl_2_ at 298 K; Figure S12: ^11^B NMR spectrum of solution [B_3_H_7_Py] in CD_2_Cl_2_ at 298 K; Figure S13: ^1^H NMR spectrum of crystal solution [B_3_H_7_Py] in CD_2_Cl_2_ at 298 K; Figure S14: ^11^B NMR spectrum of solution [B_3_H_7_Et_3_N] in CD_2_Cl_2_ at 298 K; Figure S15: ^1^H NMR spectrum of solution [B_3_H_7_Et_3_N] in CD_2_Cl_2_ at 298 K; Figure S16: Molecular packing in crystal PhNH_2_·B_3_H_7_; Figure S17: Possible intermolecular contacts in crystal PhNH_2_·B_3_H_7_; Table S1: Main crystallographic data, experimental parameters and characteristics of structure refinement for Ph_3_P·B_3_H_7_; Table S2: Selected bond lengths and bond angles in the structure of Ph_3_P·B_3_H_7_; Table S3: Main crystallographic data, experimental parameters and characteristics of structure refinement for PhNH_2_·B_3_H_7_; Table S4: Selected bond lengths and bond angles in the structure of PhNH_2_·B_3_H_7_; Table S5: Selected bond lengths and bond angles in the structure of PhNH_2_·B_3_H_7_.

## Sample Availability

Samples of the compounds are not available from the authors.
