# Supplementary material for: Nucleophilic Substitution Reactions in the [B3H8]− Anion in the Presence of Lewis Acids
Source: Molecules. 2022 Jan 24;27(3):746. doi: 10.3390/molecules27030746 (PMC8838051; doi:10.3390/molecules27030746)
Supplement: Supplementary file 1 [file molecules-27-00746-s001.zip › molecules-1481084-supplementary.pdf]

molecules

MDPI

Supporting Information

Nucleophilic Substitution Reactions in the $[B_3H_8]^-$ Anion in the Presence of Lewis Acids

Alexandra T. Shulyak [1,2,*], Evgeniy O. Bortnikov [3], Nikita A. Selivanov [1], Mikhail S. Grigoriev [4], Alexey S. Kubasov [1], Andrey P. Zhdanov [1], Alexander Y. Bykov [1], Konstantin Y. Zhizhin [1] and Nikolai T. Kuznetsov [1]

1. Kurnakov Institute of General and Inorganic Chemistry, Russian Academy of Sciences, Leninskii Pr. 31, Moscow 119991, Russia; at.shulyak@yandex.ru (A.T.S); GooVee@yandex.ru (N.A.S.); fobosax@mail.ru (A.S.K.); zhdanov@igic.ras.ru (A.P.Z.); bykov@igic.ras.ru (A.Y.B.); zhizhin@igic.ras.ru (K.Y.Z.); ntkuz@igic.ras.ru (N.T.K.)
2. Inorganic Chemistry Department, Lomonosov institute of Fine Chemical Technologies, MIREA—Russian Technological University, Pr. Vernadskogo, 86, Moscow 119454, Russia; at.shulyak@yandex.ru (A.T.S.)
3. Weizmann Institute of Science, Organic Chemistry, 234 Herzl Street, Rehovot 7610001, Israel; bortnikovevol@gmail.com (E.O.B.)
4. Frumkin Institute of Physical Chemistry and Electrochemistry, Russian Academy of Sciences, Leninskii Pr. 31-4, Moscow 119071, Russia; grigoriev@ipc.rssi.ru (M.S.G.)

* Correspondence: at.shulyak@yandex.ru

1. Experimental 2

2. NMR spectra 3

3. X-ray diffraction 11

References 16

1. Experimental

Initial reagents and solvents. $NaBH_4$, $PhCH_2Cl$, $TiCl_4$, $ZrCl_4$, $AlCl_3$, I_2, Et_3N, Ph_3P, Ph_2P-$(CH_2)_2$-PPh_2 (dppe), and Ph_3As (Aldrich) with a purity of at least 97% were used without additional purification. Triethylamine, pyridine, and aniline (Aldrich) were dried over KOH, distilled over CaH_2 (reduced pressure was used for aniline), and stored over 4Å MS. Diglyme and tetrahydrofuran (Aldrich) were passed through neutral alumina (MN aluminum oxide 90 neutral). Dichloromethane, 1,2-dichloroethane, and acetonitrile were successively washed with acid, alkali, and water, dried over calcium chloride and distilled over calcium hydride. Hexane was washed with concentrated sulfuric acid and water, dried over calcium chloride, and distilled over calcium hydride [1]. After purification, all solvents were stored over activated molecular sieves (Fluka 3Å).

[$Bu_4NB_3H_8$] was synthesized according to the method described [2] by the reaction between sodium tetrahydroborate and benzyl chloride in diglyme followed by the metathesis reaction with tetrabutylammonium bromide.

Methods. *NMR spectra* (^{11}B, ^{1}H, ^{13}C) of solutions of the studied substances were recorded on a Bruker Avance II-300 spectrometer at frequencies of 96.32 MHz, 300.21 MHz, and 75.48 MHz, respectively, with internal deuterium stabilization. Boron trifluoride etherate (^{11}B) and tetramethylsilane (^{1}H, ^{13}C) were used as external standards. *IR spectra* of the synthesized compounds were recorded on an INFRALUM FT-02 IR Fourier spectrometer (NPF AP Lumex, Russia) in the range 4000–400 cm^{-1} with a resolution of 1 cm^{-1}. Samples were prepared as KBr tablets or thin films.

X-ray single crystal diffraction was carried out at the Shared Facility Center of the Kurnakov Institute of General and Inorganic Chemistry, Russian Academy of Sciences by Grigoriev M.S. on an automatic four-circle Bruker KAPPA APEX II diffractometer with a two-dimensional detector [3] (MoK_α radiation) at a temperature of 100 K. The unit cell parameters were refined over the entire data set. The structure was solved and refined using SHELX software [4] on F^2 using all data in the anisotropic approximation for all non-hydrogen atoms. All H atoms were localized from the difference Fourier synthesis of the electron density and were refined isotropically without any restrictions. Data processing was carried out by using OLEX program [5].

Elemental analysis (C,H,N) was carried out at automatic gas analyzer CHNS-3 FA 1108 Elemental Analyser (Carlo Erba). The B determination using the ICP MS method was carried out at inductively coupled plasma atomic emission spectrometer iCAP 6300 Duo in shared experimental facilities supported by IGIC RAS state assignment FCUE "IREA" NRC "Kurchatov institute".

2. NMR spectra

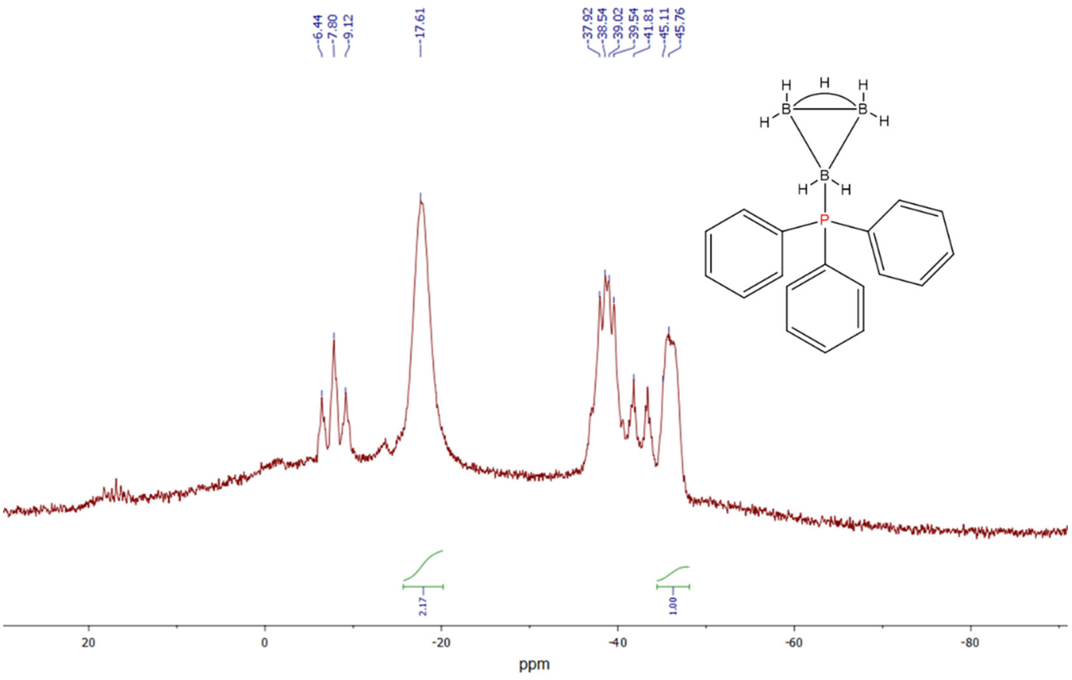

Figure S1. ^{11}B NMR spectrum of a mixture of TBA[B_3H_8], PPh_3 and $TiCl_4$ in CH_2Cl_2 30 min after the beginning of the reaction. [$B_3H_7PPh_3$] (δ -17.61 *ppm*, -45.76 *ppm*) at 298 K.

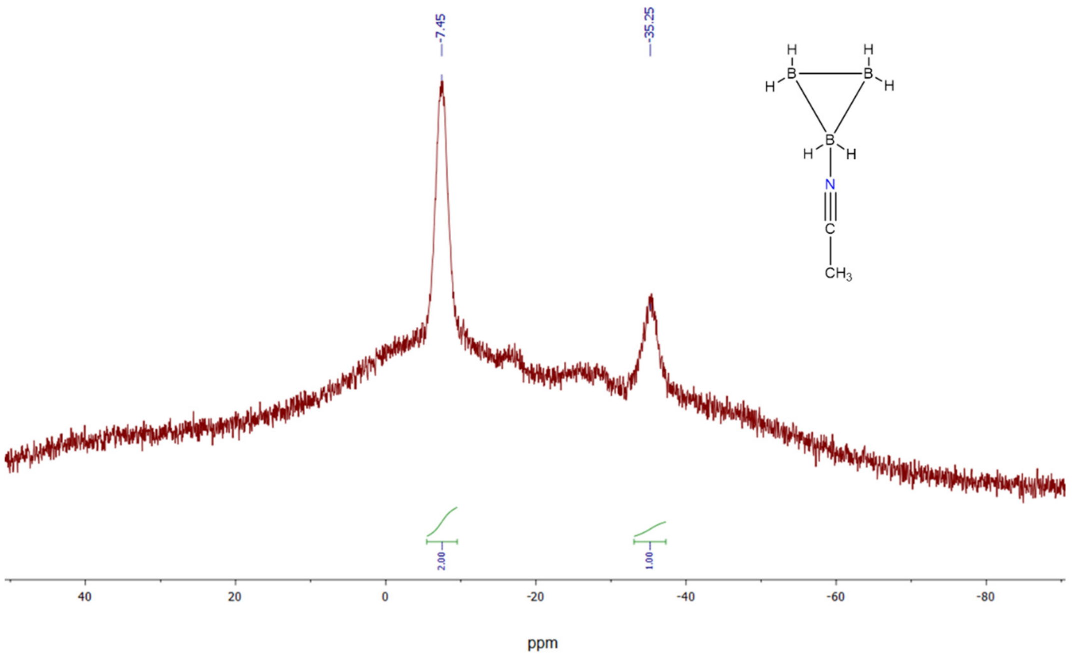

Figure S2. ^{11}B NMR spectrum of a mixture of TBA[B_3H_8], CH_3CN and $AlCl_3$ in CH_2Cl_2 2h after the beginning of the reaction. [$B_3H_7NCCH_3$] (δ -7.45 *ppm*, -35.25 *ppm*) at 298 K.

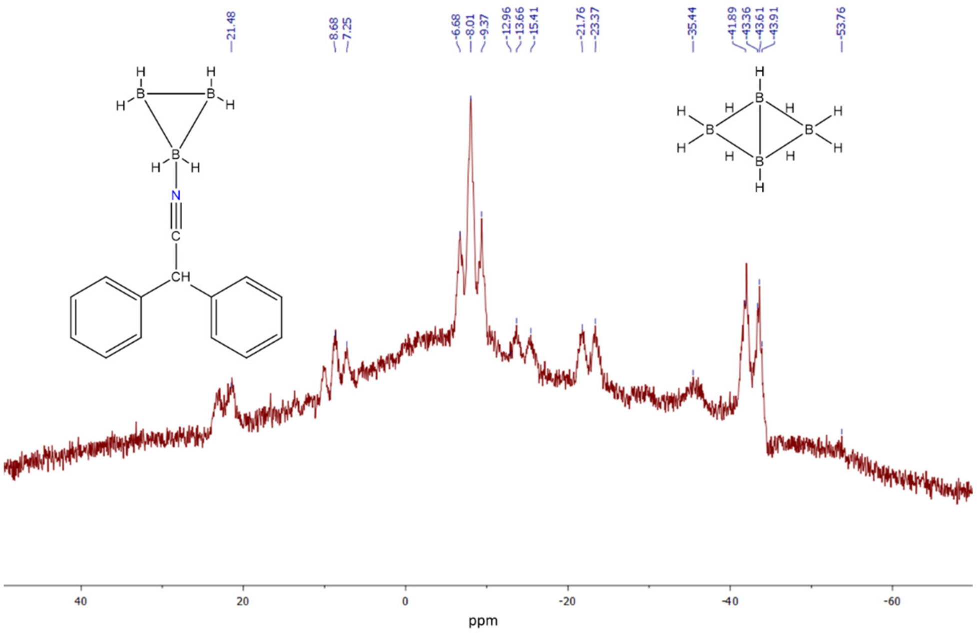

Figure S3. ^{11}B NMR spectrum of a mixture of TBA[B_3H_8], Ph_2CHCN and $AlCl_3$ in CH_2Cl_2 2h after the beginning of the reaction. [$B_3H_7NCCH_2Ph$] (δ -7.65 *ppm*, -35.44 *ppm*), [B_4H_{10}] (δ -8.01 *ppm*, -43.46 *ppm*) at 298 K.

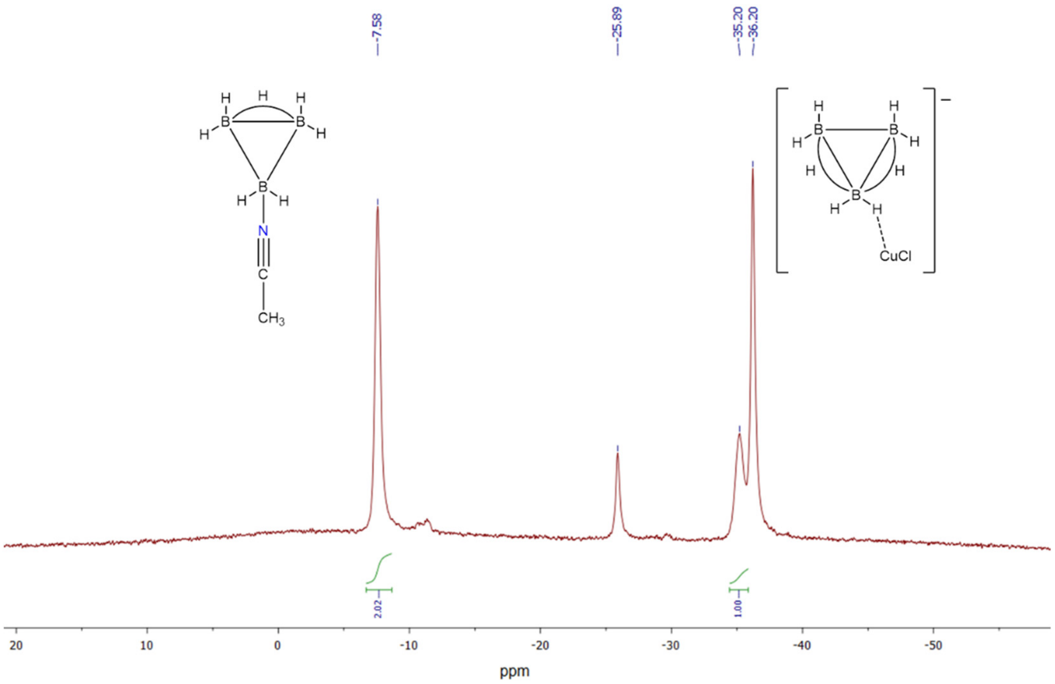

Figure S4. ^{11}B-^{1}H NMR spectrum of a mixture of TBA[B_3H_8] and CuCl in CD_3CN 30 min after the beginning of the reaction. [$B_3H_7NCCH_3$] (δ -7.58 *ppm*, -35.20 *ppm*), [BH_3NCCH_3] (δ -25.8 *ppm*), [B_3H_7HCuCl]$^-$ (δ -36.20 *ppm*) at 298 K.

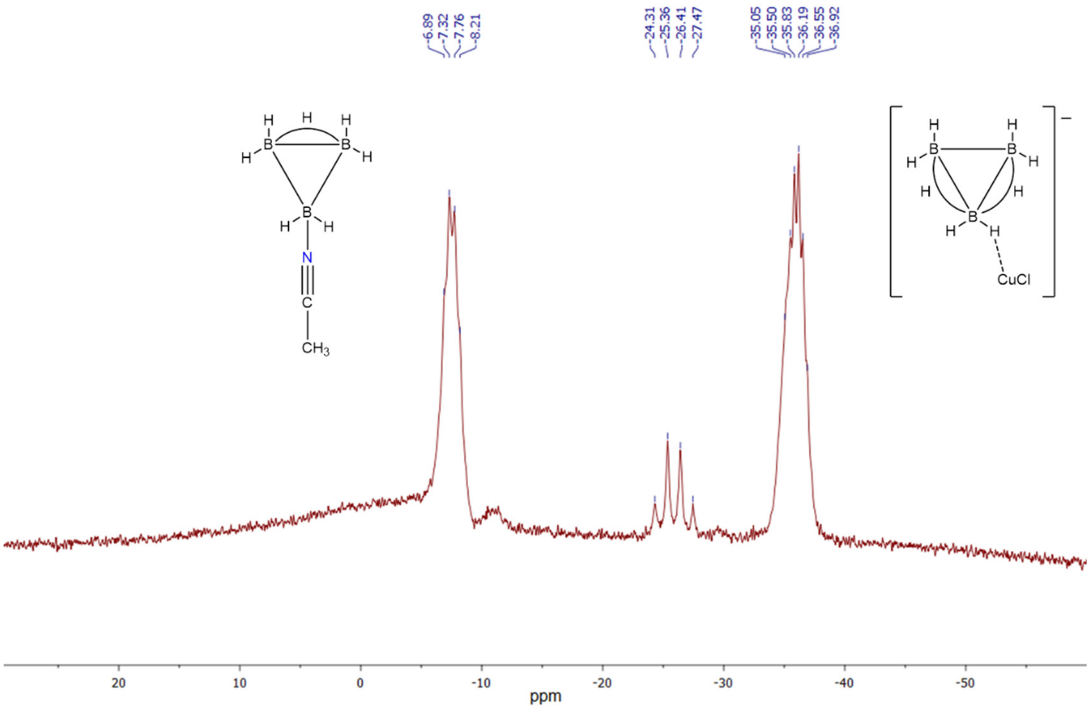

Figure S5. ^{11}B NMR spectrum of a mixture of TBA[B_3H_8] and CuCl in CD_3CN 30 min after the beginning of the reaction. [$B_3H_7NCCH_3$] (δ -7.58 *ppm*, -35.20 *ppm*), [BH_3NCCH_3] (δ -25.8 *ppm*), [$B_3H_7HCuCl]^-$ (δ -36.20 *ppm*) at 298 K.

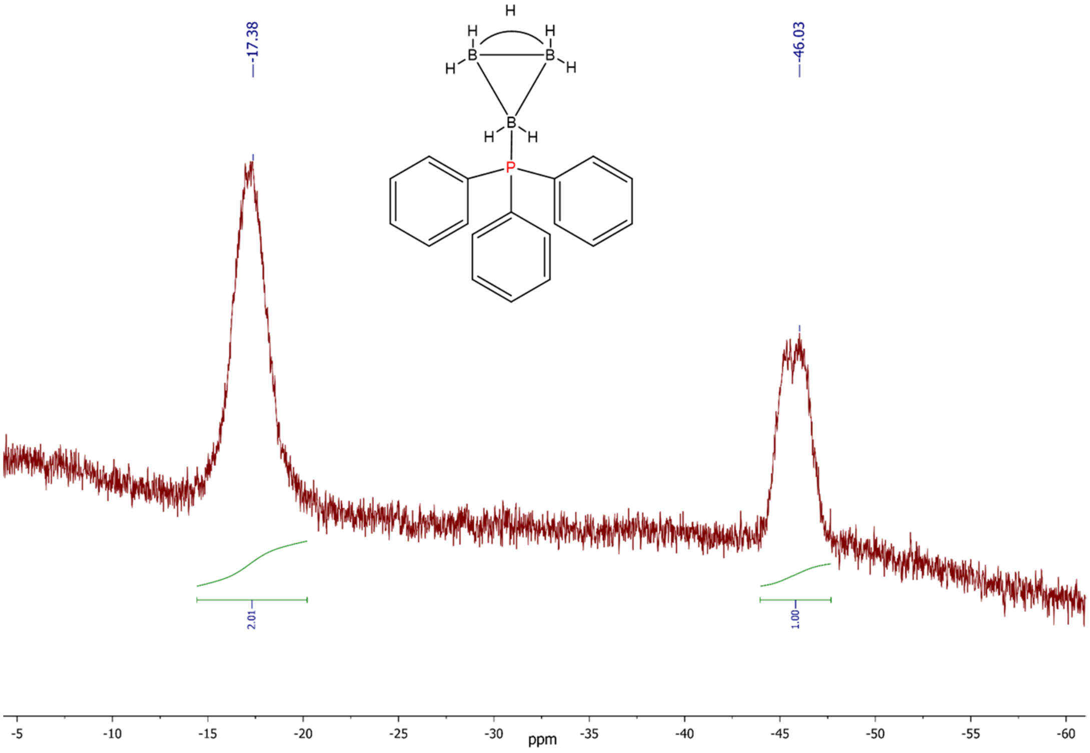

Figure S6. ^{11}B-^{1}H NMR spectrum of crystal solution [$B_3H_7PPh_3$] in CD_2Cl_2 at 298 K.

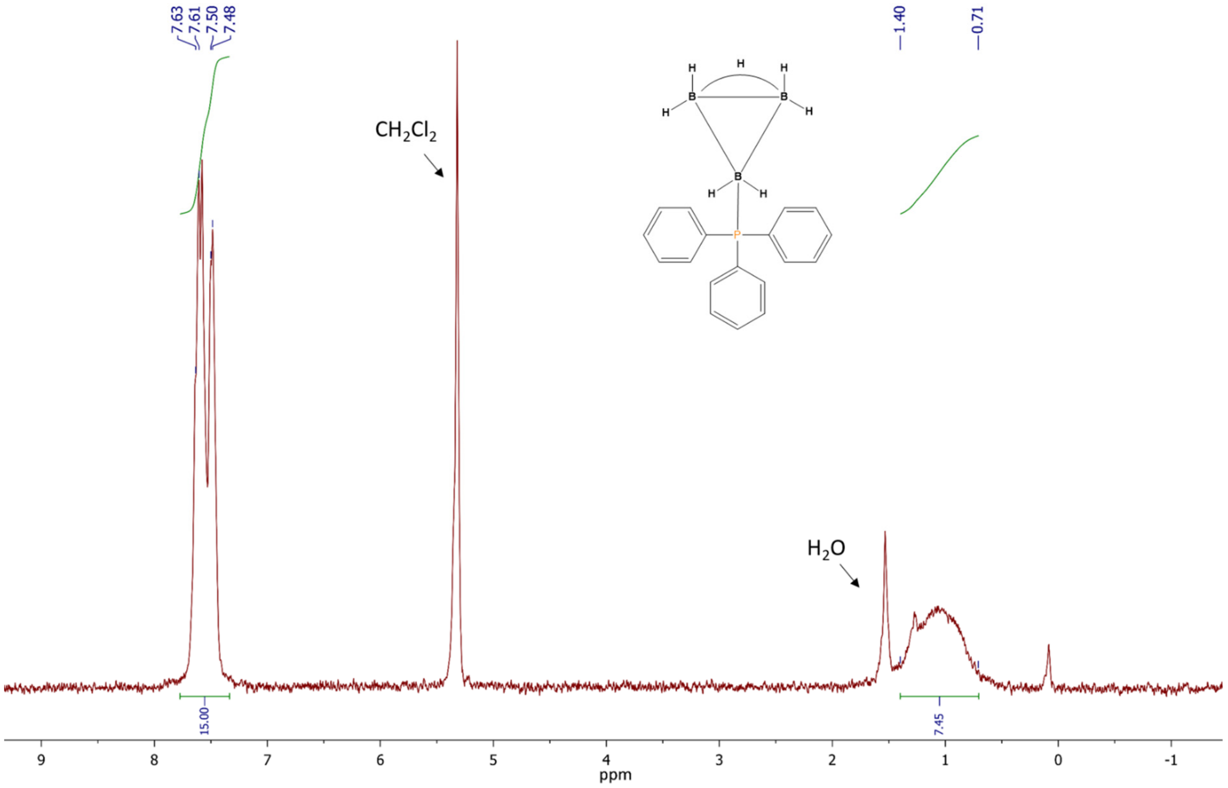

Figure S7. ^{1}H NMR spectrum of crystal solution [$B_3H_7PPh_3$] in CD_2Cl_2 at 298 K.

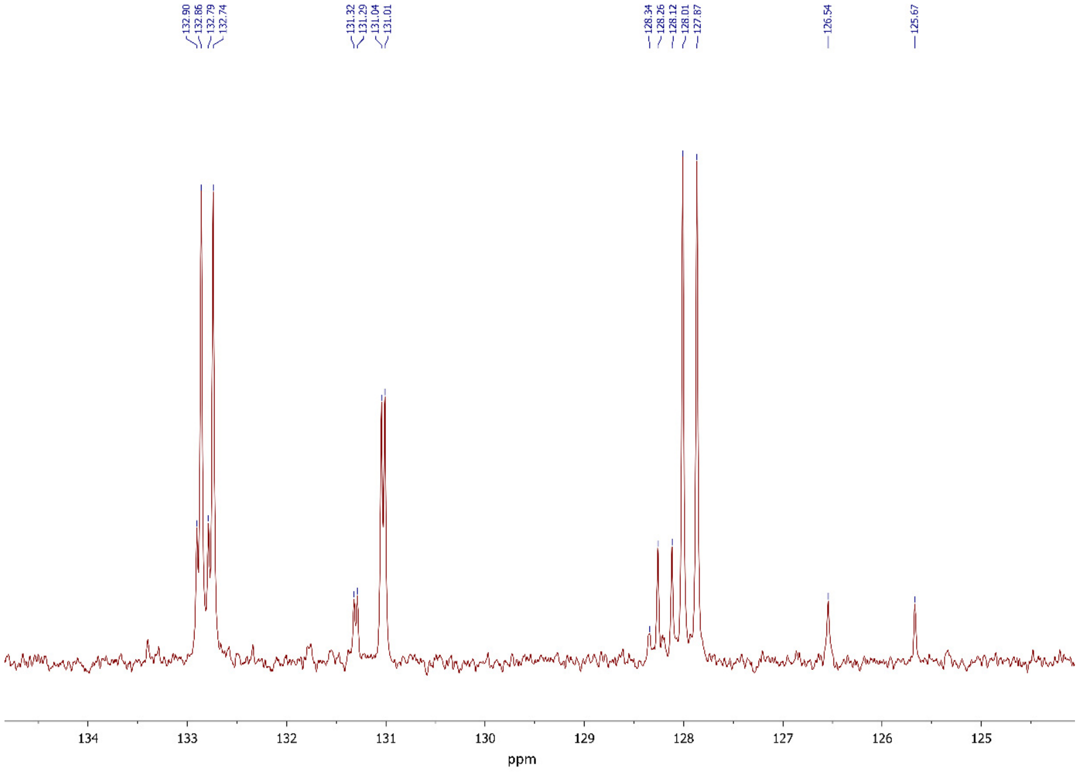

Figure S8. ^{13}C NMR spectrum of crystal solution [$B_3H_7PPh_3$] in CD_2Cl_2 at 298 K.

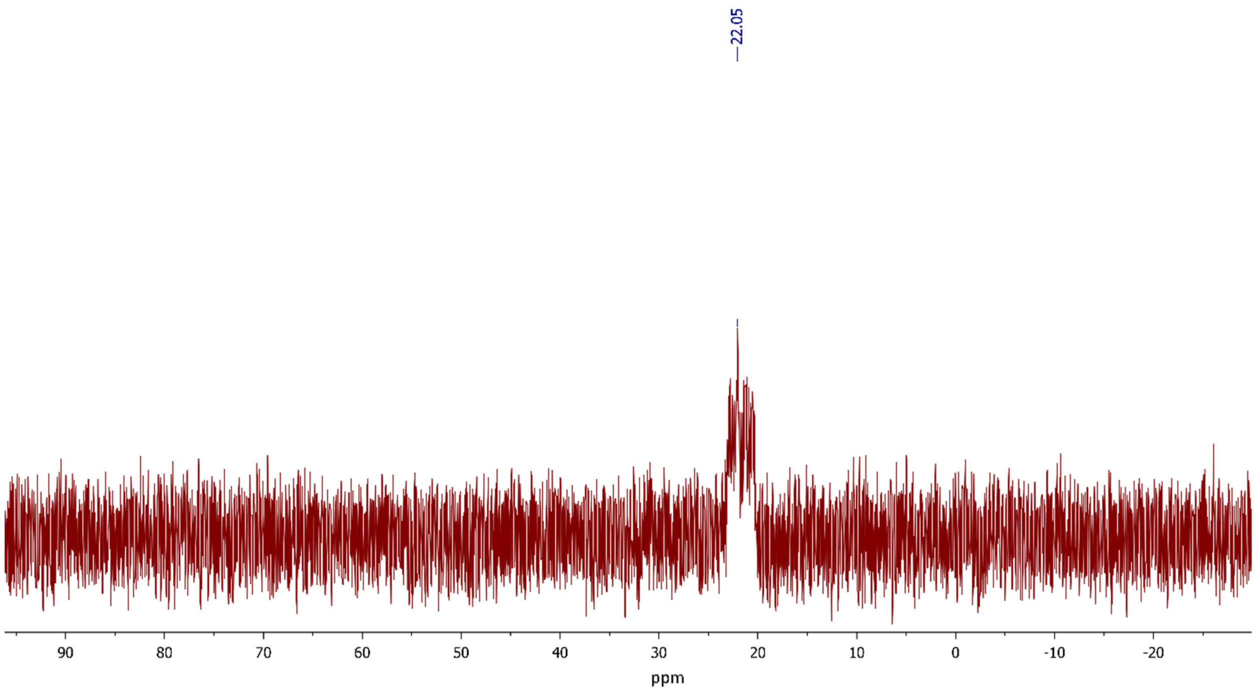

Figure S9. ^{31}P NMR spectrum of crystal solution [$B_3H_7PPh_3$] in CD_2Cl_2 at 298 K.

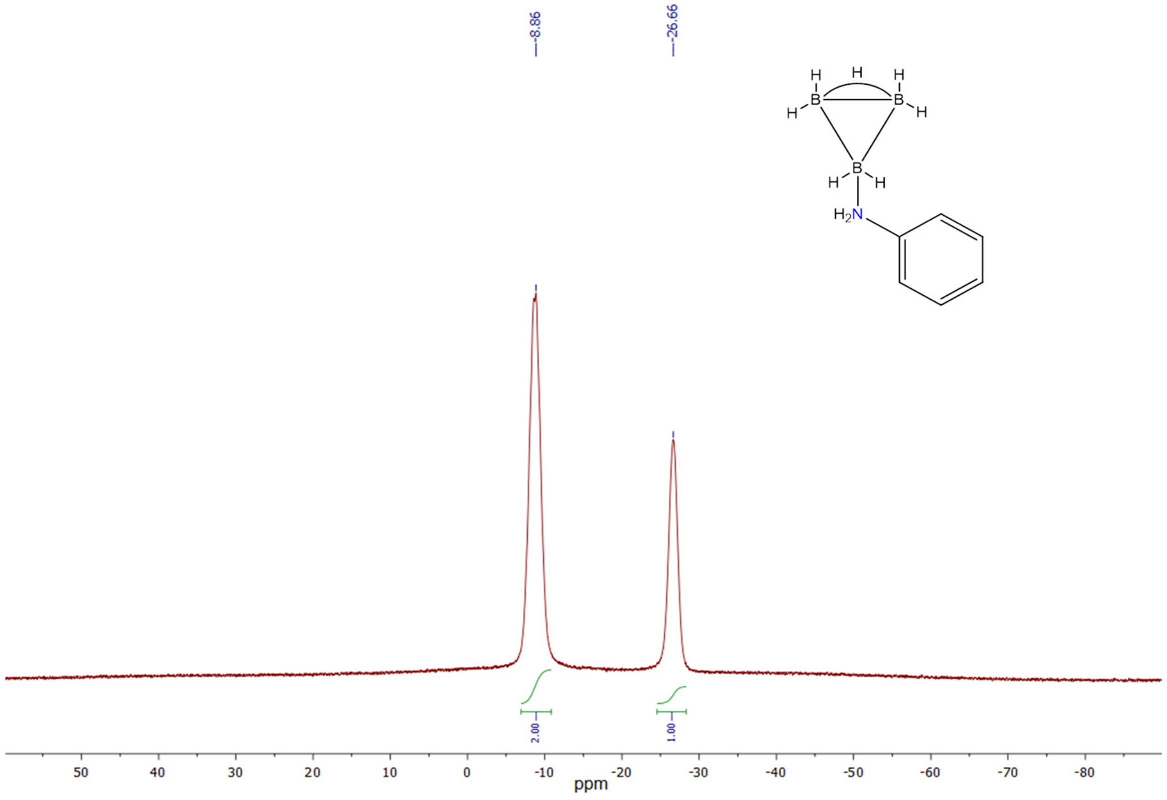

Figure S10. ^{11}B-^{1}H NMR spectrum of crystal solution [$B_3H_7NH_2Ph$] in CD_2Cl_2 at 298 K.

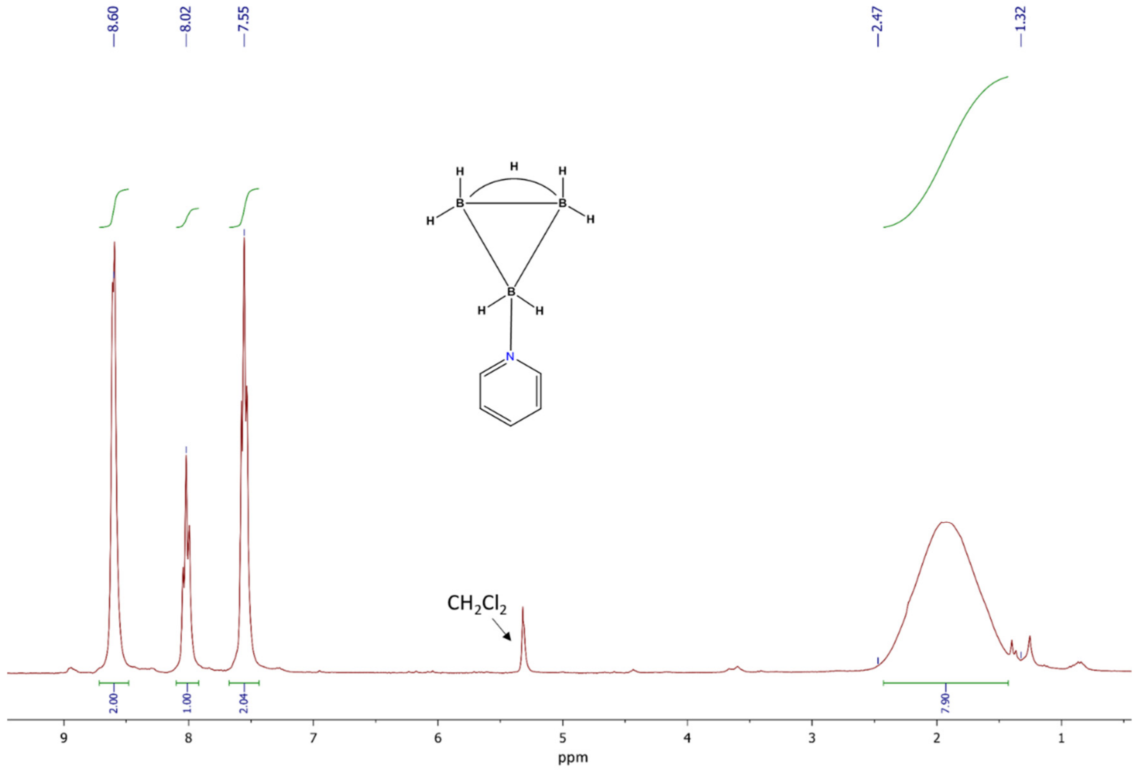

Figure S11. ^{1}H NMR spectrum of crystal solution [$B_3H_7NH_2Ph$] in CD_2Cl_2 at 298 K.

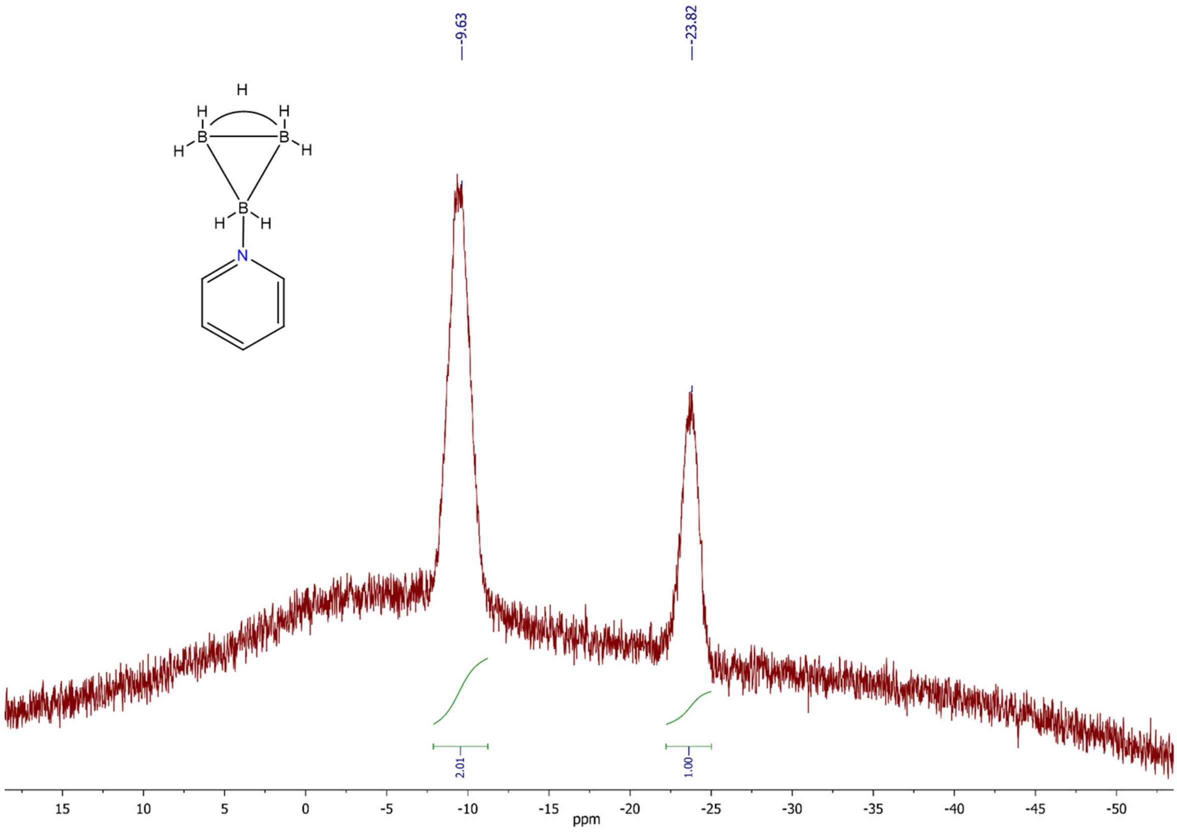

Figure S12. ^{11}B NMR spectrum of solution [B_3H_7Py] in CD_2Cl_2 at 298 K.

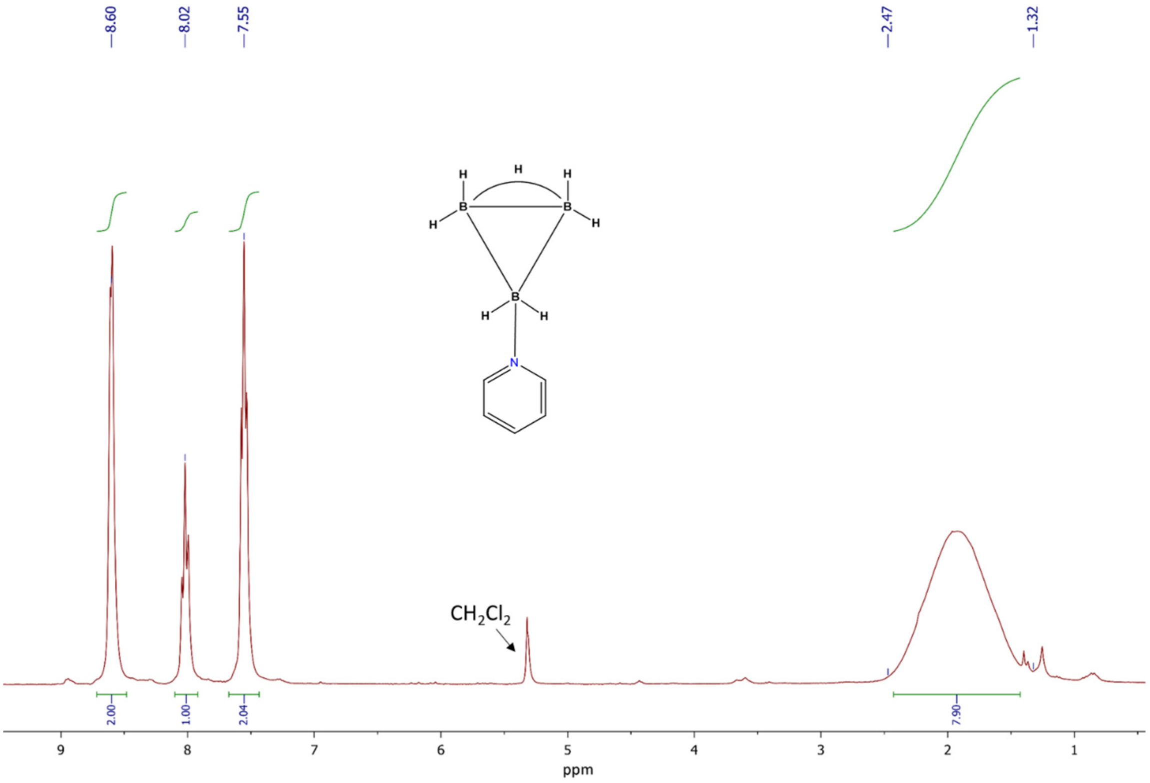

Figure S13. ^{1}H NMR spectrum of crystal solution [B_3H_7Py] in CD_2Cl_2 at 298 K.

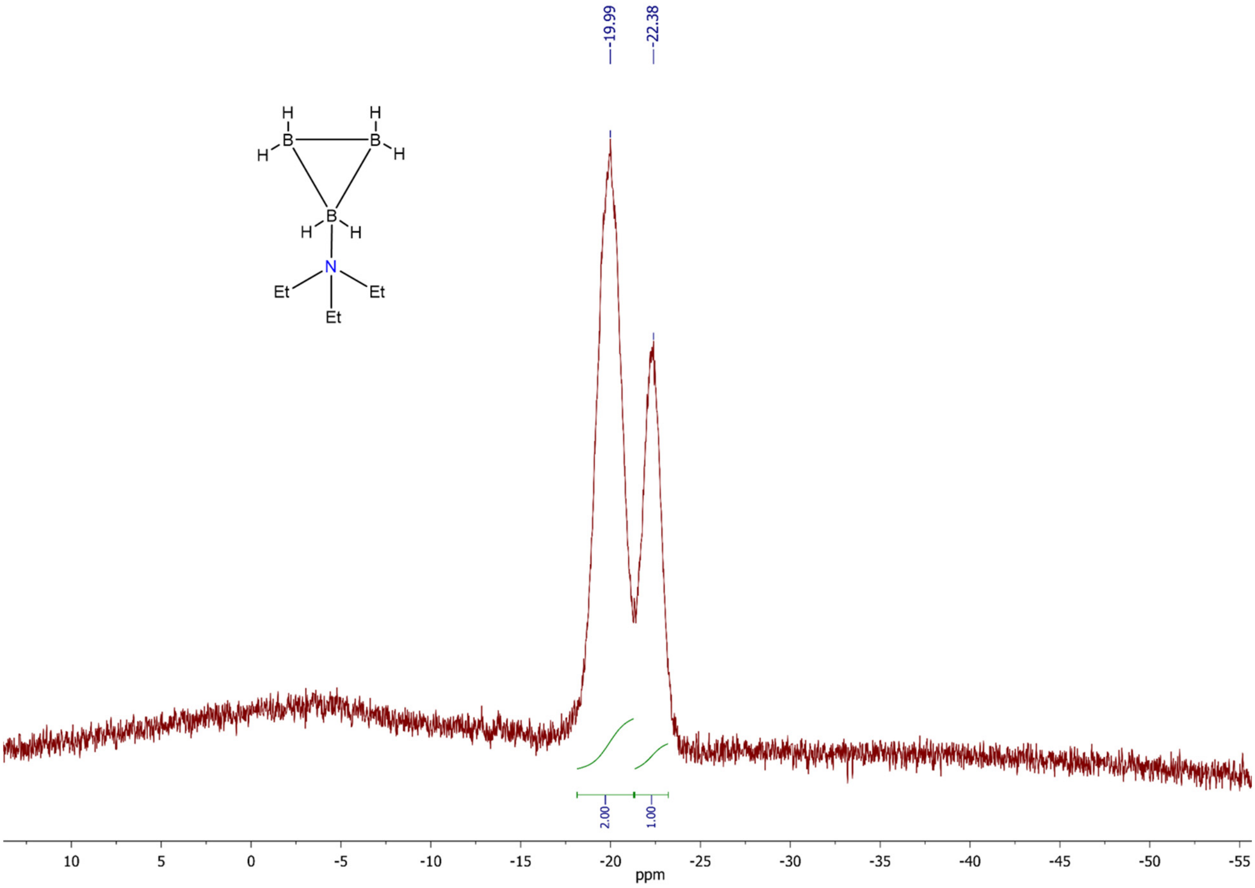

Figure S14. ^{11}B NMR spectrum of solution [$B_3H_7Et_3N$] in CD_2Cl_2 at 298 K.

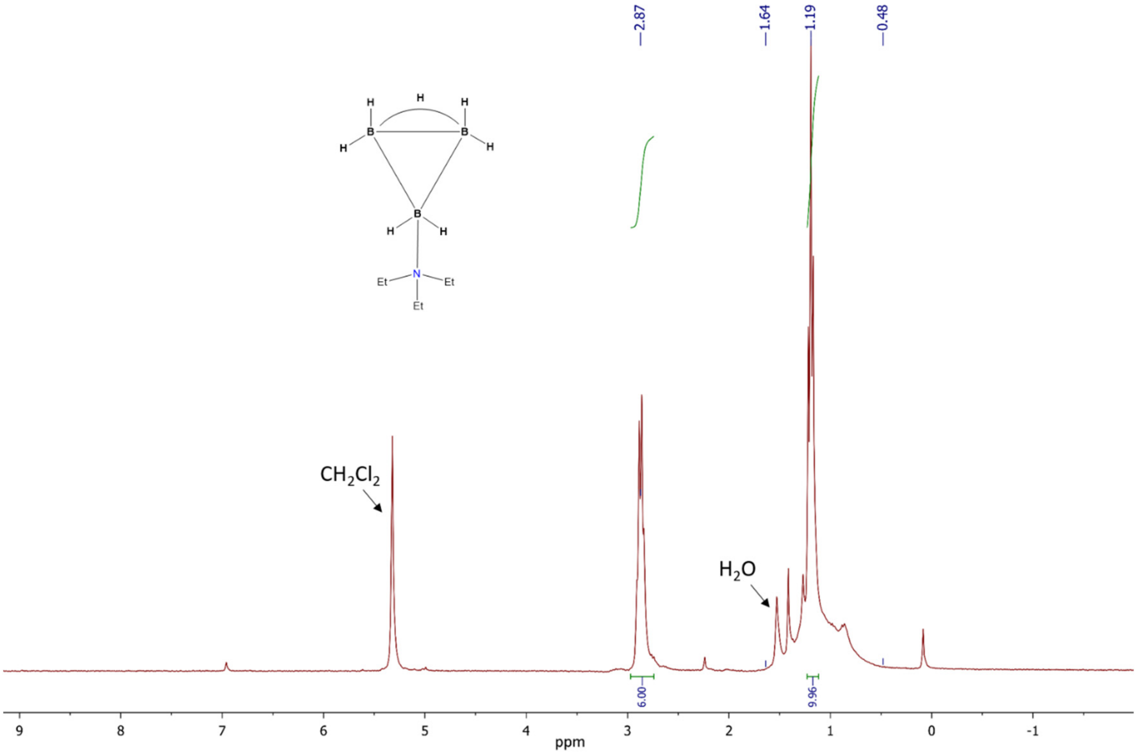

Figure S15. ^1H NMR spectrum of solution [$B_3H_7Et_3N$] in CD_2Cl_2 at 298 K.

3. X-ray diffraction

The packing in the crystal $PhNH_2{\cdot}B_3H_7$ (Figure 5, 6), apparently, occurs mainly due to the formation of dihydrogen bonds N-H···H-B. Therefore, in stacks, binding is mainly carried out through the formation of the N1-H1D···H1A-B1 interaction (2.39(4) Å), and between stacks located antiparallelly binding is realized due to the presence of a rather short contact N1-H1C···H2B-B2 (2.24(5) Å) (Figures 5 and 6). The C4-H4A···H3B-B3 distance is 2.35(4) Å; this is the way of the interaction between the aryl and borohydride fragments.

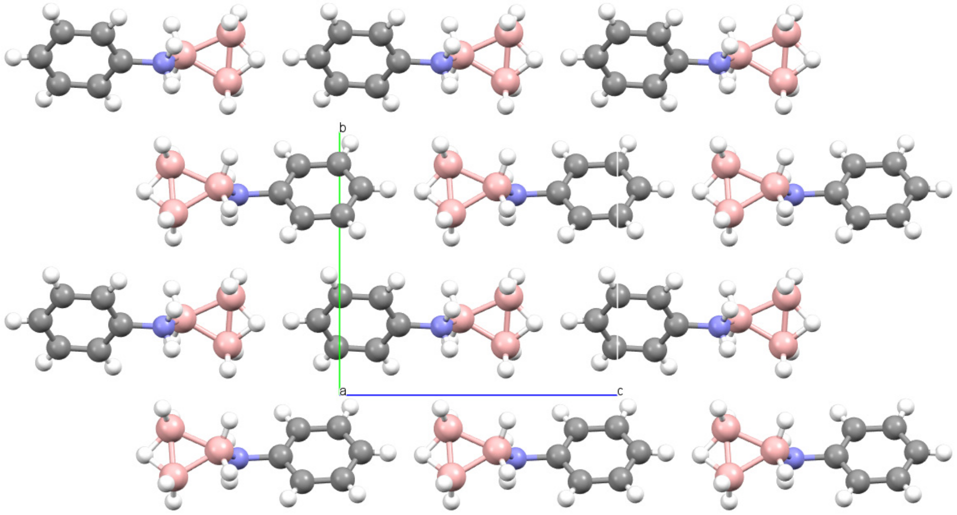

Figure S16. Molecular packing in crystal $PhNH_2{\cdot}B_3H_7$

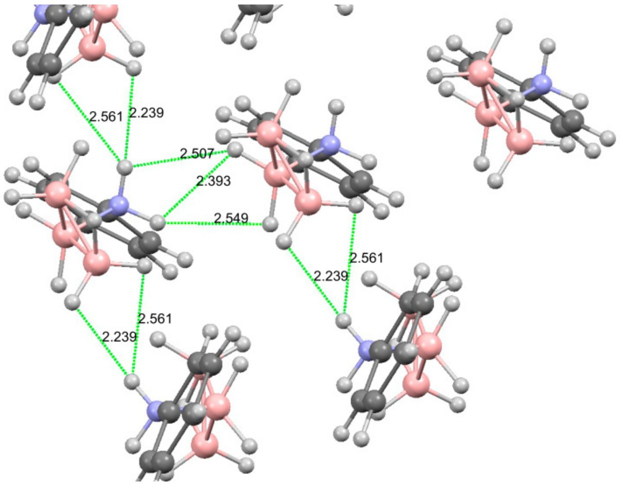

Figure S17. Possible intermolecular contacts in crystal $PhNH_2{\cdot}B_3H_7$.

Table S1. Time of the substituted products formation in the reaction of $[B_3H_8]^-$ with CH_3CN and THF

**Lewis acid**	**Time/solvent**		
	**CH_2Cl_2**	**CH_3CN**	**THF**
**$TiCl_4$**	30-40 min	30-40 min	1.5 h
**$ZrCl_4$**	30-40 min	30-40 min	1.5 h

**$HfCl_4$**	30-40 min	30-40 min	1.5 h
**$AlCl_3$**	30-40 min	30-40 min	1.5 h
**CuCl**	Has not observed (only the complex has been formed)	12 h	12 h
**$CoCl_2$**	Has not observed (only the complex has been formed)	Has not observed (only the complex has been formed)	Has not observed (only the complex has been formed)
**$ZnCl_2$**	Has not observed (only the complex has been formed)	Has not observed (only the complex has been formed)	Has not observed (only the complex has been formed)
**$MnCl_2$**	Has not observed (only the complex has been formed)	Has not observed (only the complex has been formed)	Has not observed(only the complex has been formed)

Table S2. Main crystallographic data, experimental parameters and characteristics of structure refinement for $Ph_3P{\cdot}B_3H_7$

Formula	$C_{18}H_{22}B_3P$
FW	301.75
T, K	100(2)
Crystal system	monoclinic
Space group	C2/c
a/Å	16.3802(4)
b/Å	11.5664(3)
c/Å	17.9927(4)
α/°	90
β/°	96.8160(10)
γ/°	90
V, $Å^3$	3384.80(14)
Z	8
ρ_{calc}g/cm^3	1.184
μ/mm^{-1}	0.154
F(000)	1280.0
Crystal size, mm	0.340 × 0.260 × 0.200

radiation, λ, Å	MoKα (λ = 0.71073)
2θ range, °	8.302 to 59.992
Number of reflections:<br>collected<br>independent (N) [Rint]	 18495<br>4898 [0.0264],
R1, wR2 for No	R_1 = 0.0352, wR_2 = 0.0863
*R*1, *wR*2 for *N*	R_1 =0.0800, wR_2 = 0.1404
$\Delta\varrho_{max}/\Delta\varrho_{min}$, e/Å^3	0.38/-0.22

Table S3. Selected bond lengths and bond angles in the structure of $Ph_3P{\cdot}B_3H_7$

Bond	Length, Å	Bond angle	Angle, °	Bond angle	Angle, °
P1-C11	1.812(1)	C11-P1-C21	106.03(5)	B1-B2-H23	102.2(7)
P1-C21	1.816(1)	C11-P1-C31	106.58(5)	H2A-B2-H2B	118(1)
P1-C31	1.808(1)	C11-P1-B1	108.64(5)	H2A-B2-B3	119.4(9)
P1-B1	1.926(1)	C21-P1-C31	105.83(5)	H2A-B2-H23	103(1)
B1-H1A	1.10(2)	C21-P1-B1	117.51(5)	H2B-B2-B3	117.7(8)
B1-H1B	1.10(2)	C31-P1-B1	111.62(5)	H2B-B2-H23	104(1)
B1-B2	1.804(2)	P1-B1-H1A	102.0(9)	B3-B2-H23	41.1(7)
B1-B3	1.821(2)	P1-B1-H1B	107.4(9)	B1-B3-B2	60.10(8)
B2-H1A	1.65(2)	P1-B1-B2	125.02(9)	B1-B3-H3A	111.6(8)
B2-H2A	1.10(2)	P1-B1-B3	110.91(8)	B1-B3-H3B	109.5(8)
B2-H2B	1.11(2)	H1A-B1-H1B	104(1)	B1-B3-H23	108.3(8)
B2-B3	1.779(2)	H1A-B1-B2	63.8(9)	B2-B3-H3A	121.9(8)
B2-H23	1.33(2)	H1A-B1-B3	122.6(9)	B2-B3-H3B	120.8(8)
B3-H3A	1.12(2)	H1B-B1-B2	127.4(9)	B2-B3-H23	48.2(8)
B3-H3B	1.10(1)	H1B-B1-B3	108.8(9)	H3A-B3-H3B	116(1)
B3-H23	1.17(2)	B2-B1-B3	58.79(7)	H3A-B3-H23	105(1)
		B1-B2-H2A	115.1(9)	H3B-B3-H23	107(1)
		B1-B2-H2B	112.1(8)	B2-H23-B3	91(1)
		B1-B2-B3	61.11(8)		

Table S4. Main crystallographic data, experimental parameters and characteristics of structure refinement for $PhNH_2{\cdot}B_3H_7$.

Formula	$C_6H_{14}B_3N$
FW	132.61
T, K	100(2)
Crystal system	monoclinic
Space group	$P2_1$
a/Å	4.9391(12)
b/Å	9.067(2)
c/Å	9.629(2)

α/°	90
β/°	96.807(11)
γ/°	90
V, Å^3	428.17(17)
Z	2
ρ_{calc}g/cm^3	1.029
μ/mm^{-1}	0.055
F(000)	144
Crystal size, mm	0.400×0.200×0.120
radiation, λ, Å	MoKα (λ = 0.71073)
2θ range, °	8.310 to 59.938
Number of reflections:<br>collected<br>independent (N) [Rint]	 5531<br>2415 [0.0407]
R1, wR2 for No	R_1 = 0.0460, wR_2 = 0.1141
*R*1, *wR*2 for *N*	R_1 = 0.0590, wR_2 = 0.1224
$\Delta\rho_{max}$/$\Delta\rho_{min}$, e/Å^3	0.26/-0.16

Table S5. Selected bond lengths and bond angles in the structure of $PhNH_2{\cdot}B_3H_7$

Bond	Length, Å	Bond angle	Angle, °	Bond angle	Angle, °
N1-H1C	0.88(4)	H1C-N1-H1D	100(3)	H1A-B1-H1B	108(2)
N1-H1D	0.95(3)	H1C-N1-C1	110(2)	H1A-B1-B2	129(2)
N1-C1	1.478(2)	H1C-N1-B1	110(2)	H1A-B1-B3	80(2)
N1-B1	1.608(3)	H1D-N1-C1	109(2)	H1B-B1-B2	87(1)
C1-C2	1.385(3)	H1D-N1-B1	111(2)	H1B-B1-B3	131(1)
C1-C6	1.385(3)	C1-N1-B1	115.0(2)	B2-B1-B3	55.7(1)
C2-H2C	0.93(3)	N1-C1-C2	119.2(2)	B1-B2-H2A	100(2)
C2-C3	1.392(3)	N1-C1-C6	119.6(2)	B1-B2-H2B	115(1)
C3-H3C	0.92(3)	C2-C1-C6	121.3(2)	B1-B2-H23	110(2)
C3-C4	1.387(3)	C1-C2-H2C	116(2)	B1-B2-B3	61.6(1)
C4-H4A	0.94(2)	C1-C2-C3	119.0(2)	H2A-B2-H2B	120(2)
C4-C5	1.383(3)	H2C-C2-C3	125(2)	H2A-B2-H23	105(2)
C5-H5A	1.00(3)	C2-C3-H3C	120(2)	H2A-B2-B3	123(2)
C5-C6	1.396(3)	C2-C3-C4	120.4(2)	H2B-B2-H23	107(2)
C6-H6A	0.95(3)	H3C-C3-C4	119(2)	H2B-B2-B3	116(1)
B1-H1A	1.08(3)	C3-C4-H4A	118(2)	H23-B2-B3	49(2)
B1-H1B	1.15(3)	C3-C4-C5	119.9(2)	B2-H23-B3	83(2)
B1-B2	1.847(3)	H4A-C4-C5	121(2)	B1-B3-B2	62.6(1)
B1-B3	1.830(3)	C4-C5-H5A	124(2)	B1-B3-H23	108(2)
B2-H2A	1.08(3)	C4-C5-C6	120.4(2)	B1-B3-H3A	106(2)

B2-H2B	1.11(3)	H5A-C5-C6	116(2)	B1-B3-H3B	116(1)
B2-H23	1.27(4)	C1-C6-C5	119.0(2)	B2-B3-H23	47(2)
B2-B3	1.719(4)	C1-C6-H6A	119(2)	B2-B3-H3A	123(2)
H23-B3	1.32(4)	C5-C6-H6A	122(2)	B2-B3-H3B	114(1)
B3-H3A	1.16(4)	N1-B1-H1A	109(2)	H23-B3-H3A	101(2)
B3-H3B	1.11(3)	N1-B1-H1B	109(1)	H23-B3-H3B	104(2)
		N1-B1-B2	111.0(2)	H3A-B3-H3B	120(2)
		N1-B1-B3	112.5(2)		

References

1. Bekker H. *Organicum: V2.* Moscow, Mir; 1992.
2. Bykov, A.Y; Mal, N.N; Generalova, N.B; Zhizhin, K.Y; Kuznetsov, N.T; Reactions of Sodium Tetrahydroborate with Alkyl and Aryl Halides : A New Approach to the Synthesis of $[B_3H_8]^-$ Anions and $[B_{12}H_{12}]^{2-}$. **2013**, 58(11), 1321-1323. doi:10.1134/S003602361311003X
3. Bruker AXS Inc. APEX2 (V. 2009, 5-1), SAINT (V7.60A), SADABS (2008/1).
4. Sheldrick, G.M; A short history of *SHELX*. *Acta Crystallogr Sect A Found Crystallogr*. **2008**, 64(1), 112-122. doi:10.1107/S0108767307043930
5. Dolomanov, O.V.; Bourhis, L.J.; Gildea, R.J.; Howard, J.A.K.; Puschmann H. OLEX2: A complete structure solution, refinement and analysis program. *J Appl Cryst*. 2009;42:339-341.
